# Insights into the Multiscale Lubrication Mechanism of Edible Phase Change Materials

**DOI:** 10.1021/acsami.2c13017

**Published:** 2023-01-12

**Authors:** Siavash Soltanahmadi, Michael Bryant, Anwesha Sarkar

**Affiliations:** † Food Colloids and Bioprocessing Group, School of Food Science and Nutrition, University of Leeds, LeedsLS2 9JT, U.K.; ‡ Institute of Functional Surfaces, School of Mechanical Engineering, University of Leeds, LeedsLS2 9JT, U.K.

**Keywords:** soft tribology, coalescence, aqueous lubrication, friction coefficient, oral processing, papillae, chocolate, saliva, tongue-like surface

## Abstract

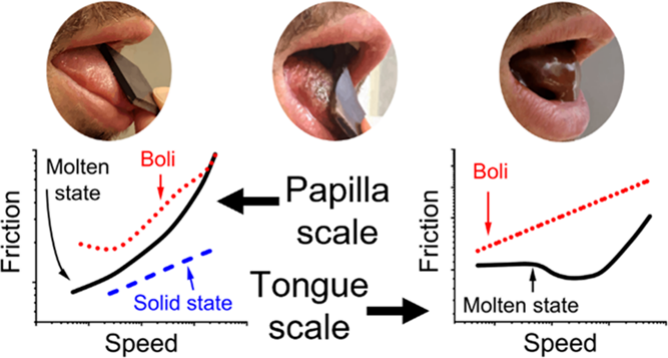

Investigation of a lubrication behavior of phase change materials (PCM) can be challenging in applications involving relative motion, *e.g.*, sport (ice skating), food (chocolates), energy (thermal storage), apparel (textiles with PCM), etc. In oral tribology, a phase change often occurs in a sequence of dynamic interactions between the ingested PCM and oral surfaces from a *licking* stage to a *saliva-mixed* stage at contact scales spanning micro- (cellular), meso- (papillae), and macroscales. Often the lubrication performance and correlations across length scales and different stages remain poorly understood due to the lack of testing setups mimicking real human tissues. Herein, we bring new insights into lubrication mechanisms of PCM using dark chocolate as an exemplar at a single-papilla (meso)-scale and a full-tongue (macro) scale covering the solid, molten, and saliva-mixed states, uniting highly sophisticated biomimetic oral surfaces with *in situ* tribomicroscopy for the first time. Unprecedented results from this study supported by transcending lubrication theories reveal how the tribological mechanism in licking shifted from solid fat-dominated lubrication (saliva-poor regime) to aqueous lubrication (saliva-dominant regime), the latter resulted in increasing the coefficient of friction by at least threefold. At the mesoscale, the governing mechanisms were bridging of cocoa butter in between confined cocoa particles and fat coalescence of emulsion droplets for the molten and saliva-mixed states, respectively. At the macroscale, a distinctive hydrodynamic viscous film formed at the interface governing the speed-dependent lubrication behavior indicates the striking importance of multiscale analyses. New tribological insights across different stages and scales of phase transition from this study will inspire rational design of the next generation of PCM and solid particle-containing materials.

## 1 Introduction

Phase change materials (PCM) are attractive classes of materials with broad applications ranging from the energy sector (thermal management, battery applications, *etc.*) and photonic to the neuroinspired computing where often a change between states of matter involves a transition between amorphous and crystalline phases.^1^ The phase transition can occur when the source of heat to propel the phase change is tribological stresses or when a PCM is exposed to environmental factors in an application. Examples of the former and latter are skiing blades transforming snow to water and oral processing that transforms solid macroscopic food structures to molten mixtures of the food with the saliva (*i.e.*, a nature-engineered biolubricant).^2^ In the case of skiing, the kinetics of phase transition due to the tribological contact determines the right surface roughness on ski blades^3^ and the thickness of a film of the molten ice determines the extent of frictional forces.^4^ In ingested PCM systems, where phase changes are abundant owing to processing at industrial scale (*i.e.*, manufacturing of a chocolate) as well as the biological exposure upon consumption (*e.g.*, temperature, salivary enzymes, *etc.*), mechanistic studies on the multiscale lubrication behavior of edible PCM are currently limited.

Chocolate is a classic edible PCM, which mainly consists of suspended particles (cocoa solid and sugar crystals) in crystalline cocoa butter. The oral perception of chocolates starts with either biting or licking. The biting often correlates with the bulk properties of chocolates and involves tooth–chocolate contacts,^5^ largely falling into the area of fracture mechanics and is relatively well-studied.^6^ The concentration, size distribution, and shape of solid particles of chocolates along with the type and concentration of emulsifier (*e.g.*, soy lecithin and polyglycerol polyricinoleate) influence the bulk properties, flow characteristics, and lubrication behavior of chocolates in mouth, impacting the gustation (*i.e.*, the primary taste perception).^5,7−9^ Thinking of the stages of oral processing, the licking starts with a direct contact between the tongue and a solid chocolate followed by a gradual phase transition of the chocolate from a crystalline solid to a continuous molten fat phase containing suspended particles of cocoa and sugar. The molten chocolate is eventually mixed with the biological fluid, *i.e.*, saliva,^5^ which gradually dissolves the sugar crystals.^10^ The licking process, *i.e.*, the solid-lubrication behavior of chocolates, remains principally unexplored.

The later stages of chocolate in mouth (*i.e.*, molten chocolate and saliva-mixed) have been studied through sensory trials and rheological and tribological methods. Tribology has been proven to be an enabling field of study where other conventional methods (*i.e.*, rheology, *etc.*) have failed to provide understanding of the tactile sensation of food systems from emulsions to semisolid foods, and deciphering food–saliva interactions.^11−14^ Consequently, oral tribology has contributed to informed design of healthy foods as well as tailored food for vulnerable populations.^11,15−17^ In addition to flavor-induced retronasal characteristics of chocolates, the temporal profile of chocolates in a myriad of food–mouth interactions correlates to the frictional behavior of chocolates, which are translated to sensory attributes such as smoothness, coarseness, grittiness, etc.^11−13,15,16^

Attempts have been made to understand often unpleasant sensory attributes of dark chocolates (*e.g.*, grittiness, pasty, mouth-coating) through tribological studies of molten chocolates and their mixtures with saliva.^8,9,18−20^ These studies have provided invaluable information on the mouth-feel of chocolates, albeit using materials (*e.g.*, smooth polydimethylsiloxane surfaces) or conditions that are far from real biophysical characteristics of tongue–palate contact. As recently evidenced with edible polymers,^21−23^ the distinctive micropapillated architecture of real human tongue may have pivotal tribological consequences, which is poorly understood to date in PCM. Of more importance, the intricate nature of chocolates (as a PCM) and their interactions with saliva across solid to saliva-mixed stages have not been explored from a multiscale perspective to date.

With our recently fabricated three-dimensional (3D) biomimetic tongue-like surface,^21^ which emulates the topography, deformability, and wettability of a real human tongue surface, herein, we took a multistage approach representing licking to saliva-mixed stages to decipher the lubrication mechanisms of dark chocolates at a *tongue-scale* as well as at a *single-papilla*-scale in orally relevant contact conditions. For the first time, we investigated solid lubrication behavior of chocolates (*i.e.*, before its phase transition) and exploited an *in situ* tribomicroscopy to provide insights into different stages of oral processing of chocolates, supported by theoretical considerations. In this study, we demonstrate that the classical lubrication theories have failed to fully explain the complex tribological behavior of chocolates. The scale-dependent interplays of solid lubricity, aqueous lubrication, hydrodynamic forces, and particle entrainment as a function of the stage of processing and speed are discussed. This study offers a novel pathway to design edible PCM such as the one with a *gradient design* to contain a higher degree of cocoa butter at the chocolate interface, showing a promising prospect to produce low-calorie dark chocolates with pleasant mouth-feel. Therefore, the fundamental multiscale insights provided by this work can facilitate engineering metamaterials, which undergo a phase transition when subjected to tribological stresses and application-specific factors (*e.g.*, saliva in this study).

## 2 Materials and Methods

### 2.1 Materials

#### 2.1.1 Chocolates and Their Mixtures with the Model Saliva

Four commercial chocolate samples (Lindt Excellence, Lindt & Sprüngli, U.K.) were used in this study containing 70–99 wt % cocoa content. The compositions of chocolates as stated on the ingredient list of the chocolate labels are provided in Tables 1 and S1. The chocolates were purchased from online grocery shops on a single order to minimize the batch-to-batch variation. All chocolate samples in this study fall into the “*dark chocolate*” category. This close-packed range (70–99 wt %) was intentionally considered to critically assess the capability of testing apparatus used in this study, to mainly distinguish the chocolates’ frictional behavior.

**Table 1:** Composition of Chocolate Samples^a^

chocolate samples (%)	cocoa content (wt %)	total fat (sat) (wt %)	total sugar (wt %)
70	70	41 (24)	29
85	85	46 (28)	11
90	90	55 (30)	7
99	99	51 (31)	1

a The concentration of total fat, saturated fat, cocoa, and sugar in the chocolate samples as per the manufacturer.

The model saliva (S) was prepared based on a previously reported protocol^24^ using porcine gastric mucin (PGM Type II, Sigma-Aldrich, M2378) comprising MUC5AC and MUC6 mucins. A solution of PGM at 3 wt % was used to obtain the viscosity values close to that of the human saliva.^24,25^ All other chemicals used to prepare S were analytical grade and are listed in Table S2. The chemicals were used as received, without any further purification and were dissolved in Milli-Q water (resistivity of 18.2 MΩ·cm at 25 °C, Millipore Corp., Bedford, MA) and finally the pH was adjusted to 7.0 using an aqueous solution of 1.0 M NaOH. The blends of chocolate and the model saliva (referred to as chocolate-S hereafter) were prepared immediately before the measurements at a ratio of 1:1 wt % and at 37 °C; each blend weighting ∼30 g in the liquid state (see S3 for more information).

#### 2.1.2 Testing Specimens

Polydimethylsiloxane (P) probes and discs for tribological tests at the single-papilla-scale were prepared using a silicone Sylgard 184 Kit (Dow-Corning, Michigan). The P monomer solution and its curing agent were mixed at a weight ratio of 10:1 according to the recommendation of the supplier. The P probes were fabricated through casting into 96-well round-bottom cylindrical microplates (Corning 353077, 353227―radius of curvature of 3.1 mm). The P discs were produced using a polytetrafluoroethylene cylindrical mold measuring 46 mm in diameter and 4 mm in thickness. The casted polydimethylsiloxane solutions were cured at 50 °C for 24 h to obtain probes and discs with a Poisson’s Ratio (ν) and Young’s modulus (*E*) of 0.49 and 2.1 MPa, respectively.^26^ A borosilicate planoconvex lens (LA1470―N-BK7, Thorlabs, Inc.) with ν, *E*, and radius of curvature (*r*) of 0.206, 82 GPa, and 6.2 mm, respectively, was used as a glass (G) probe for the tribological tests. Both G and P surfaces had roughness (*R*_a_) values < 20 nm.

The tongue-mimicking elastomeric surfaces containing two types of papillae on a human tongue (*i.e.*, filiform and fungiform) were produced based on our previously published work.^21,23^ A numerical algorithm was developed using Matlab programming software (MathWorks) to generate random coordinates for 200 filiform-mimicking cylindrical rods of 250 μm height and 350 μm diameter, along with 20 fungiform-mimicking hemispheres of 500 μm height and 1000 μm diameter (per unit cell of 1 cm^2^) on a master mold based on a spatial Poisson point distribution, defined by eq 1
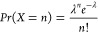
1where *X*, the random variable, indicates the number of coordinates in a defined area and λ describes the rate of occurrence (*i.e.*, the expected number of papillae in a unit area).

The drawing for the master mold (negative impression) that simulates a real human tongue^21,23^ was generated using a solid modeling software (AutoCAD, Autodesk, 2020) and was fabricated using a 3D Printer (EnvisionTEC, Dearborn) on an acrylic resin (Perfactory HTM140). Ecoflex 00-30 (Smooth-On) silicone elastomer was mixed with a Sorbitan oleate surfactant (Span 80, Sigma-Aldrich, Dorset, U.K.) at 0.05 wt % and degassed (Intertronics, Thinky ARE-250) immediately before casting into the master mold that was pretreated with a solution of poly(vinyl alcohol). The casted Ecoflex elastomer was cured at room temperature (22 °C) for 5 h and peeled off the master mold. The replica (positive impressions) was decontaminated through sonication in iso(propyl alcohol) (IPA) and deionized (DI) water for 10 min each. This soft-lithographic technique generated tongue-mimic surfaces containing randomly distributed filiform and fungiform papillae conforming to the characteristics of a real human tongue in terms of its topography (size and distribution of papillae), wettability, and mechanical properties.^21^ The tongue-mimic elastomers had ν and *E* of 0.49 and 130 kPa, respectively.^21,22^

### 2.2 Methods

#### 2.2.1 Contact Mechanics and Estimation of Fluid-Film Thickness

The Hertzian contact theory for a contact of a hemisphere with a half-space plane (a point contact) was used to estimate the maximum contact pressure (*P*_max_), the contact radius (*r*_contact_), and the indentation depth (δ).^11^ The governing equations are as follows^11^
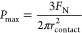
2
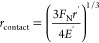
3
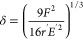
4where *F*_N_, *r*′, and *E*′ are the normal load, effective radius of curvature in the direction of entrainment of a fluid film (*i.e.*, molten chocolates and their mixtures with S), and the equivalent modulus of elasticity of contacting bodies (G or P or tongue-mimic), respectively, which are given as below^11^
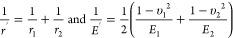
5where (*r*_1_, *r*_2_), (*E*_1_, *E*_2_), and (ν_1_, ν_2_) are the radii of curvature, the elastic moduli, and Poisson’s ratios, respectively, of the two contact bodies (1 and 2). The δ for a single hemisphere-shaped fungiform on the biomimetic tongue elastomers at the tongue-scale experiments was estimated to be <200 μm (less than the height gap between the fungiform and the filiform) and therefore, a direct contact of filiform papillae on the surface of the tongue-mimic samples with the counterbody is not expected (see Figure S1for more information).

The thickness of the hydrodynamic fluid film was estimated for chocolate samples in the molten (*i.e., initial mastication*) stage to obtain insights into whether the cocoa/sugar particles entrained into the contact interface. The following equation for the iso-viscous-elastic regime was used to calculate the minimum film thickness (*h*_min_) at the contact interface^11,15,27^
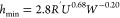
6where *U* is the dimensionless speed parameter , *W* is the dimensionless load parameter , η_∞_ is the viscosity (η) of the fluid film at the tribologically relevant high shear rates (γ̇), and *u* is the entrainment speed. The η_∞_ often is taken as the limiting high-shear viscosity of the fluid in rheological measurements (the second plateau in the η–γ̇ graphs) and is a measure of the hydrodynamic forces generated by the fluid film during tribocontacts.^11,28^ The majority of the tribological results in this study are presented as μ – *u* and μ – η_∞_ × *u* (*i.e.*, the product of the limiting high-shear viscosity and *u*) graphs. The latter is presented to normalize the μ – *u* plots to the influence of viscous forces of the fluid film in full-film lubrication regimes (discussed in the Results section).^23,28^

#### 2.2.2 Particle Size Measurements

To evaluate the size of the solid particles in the chocolate samples, each chocolate was dispersed in sunflower (SF) oil at a weight ratio of 1:20 (Tesco, U.K.) at 25 °C using an ultrasonic bath followed by laser scattering. The particle size distribution (PSD) of the chocolate samples was assessed through the Mie theory of light scattering using a Mastersizer 3000 (Malvern analytical, U.K.) over a broad size measurement range between 0.01 and 10,000 μm. An absorption index of 0.100 and refractive indices of 1.590 and 1.469 for the particles and the SF oil were assumed, respectively.^8^ The measurements were carried out at 25 °C and the laser obscuration was maintained below 10–12%. The PSD curves were deconvoluted by Gaussian distribution function using OriginPro to obtain two peaks.^8^

#### 2.2.3 Bulk Rheological Measurements

A modular compact rheometer (MCR-302, Anton Paar, Austria) was used to measure the apparent viscosity (η) of the chocolate and chocolate-S samples via a double gap (DG 27, bob outer diameter 27.0 mm, cup outer diameter 29.2 mm, gap size ∼ 1 mm) and a plate-on-plate (PP50, a 50 mm top plate on 60 mm bottom plate, gap size 1 mm) geometry, respectively, at 37 °C—*i.e.*, oral physiological temperature. A double gap was selected for the chocolate samples due to slippage of the chocolates from the measurement gap in the plate-on-plate or cone-on-plate geometries at high γ̇ (depending on the chocolate, typically after 100 s^–1^). The measured η values using the plate-on-plate geometry showed no significant difference to those obtained with the double-gap geometry where there was no slippage (see Figure S2).

The η results were measured from a shear rate (γ̇) of 0.1 to a maximum of 2000 s^–1^ in an incremental logarithmic order. Five data points were logged within each decade of the γ̇ range with an allocated 30 s window for each data logging to ensure stress stability. The measurements for the chocolate-S mixtures were comprised of a preshear step at γ̇ = 500 s^–1^ for 120 s to ensure a homogeneous mixture. The samples were sonicated for 5 min and allowed to rest at the measurement gap for 30 s to reach a steady state immediately before the measurements. A thermocontrol hood was used to minimize the influence of local chocolate solidification during the measurements.

#### 2.2.4 Confocal Scanning Laser Microscopy

A Zeiss LSM 880 inverted confocal microscope (Carl Zeiss MicroImaging GmbH, Jena, Germany) was used to examine the microstructure of 70% (shown in Figure S3) and 90% chocolates and their mixtures with S in the pristine condition (as prepared) and after the triboshear measurements. A solution of Nile red in dimethyl sulfoxide (1 g L^–1^) was added to the samples at a final concentration of 0.02 g L^–1^ to stain the oil droplets. Immediately after sample preparation (melting and mixing) or tribotesting, the samples were casted into a concave confocal microscope slide followed by staining while samples were in a molten state. Therefore, the samples were not exposed to post-processing steps (*e.g.*, remelting, heating) prior to imaging. All images were captured with an oil-immersion 40× Plan-Apochromate at 2% laser power and on a two-track mode exciting the Nile red at 514 nm and autofluorescent chocolate flavonoids at 488 nm.

#### 2.2.5 Tribological Performance

**Figure 1:**
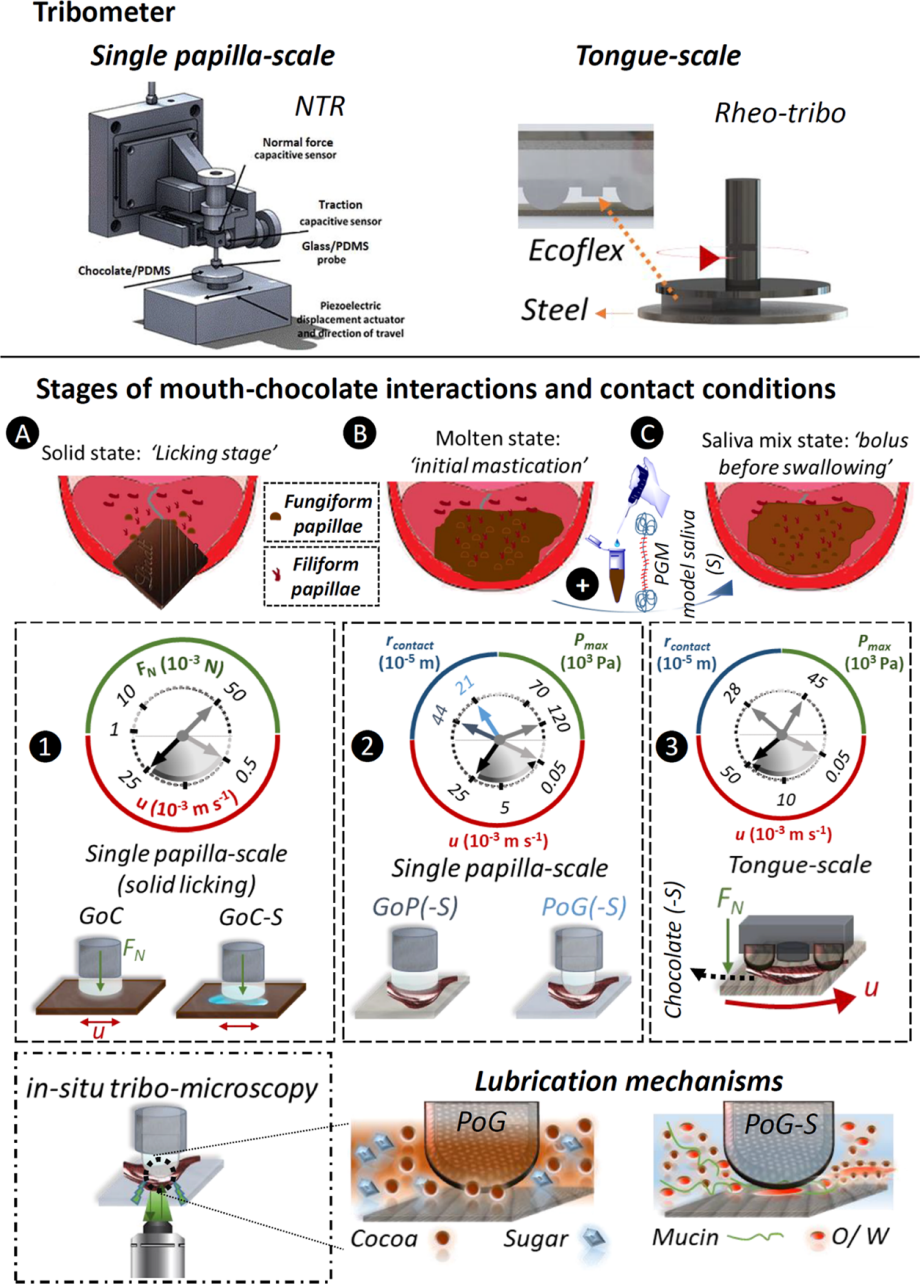
Schematic illustration of the tribological setup across scales. Two schematically shown tribometers were used to emulate a contact of a single-papilla on a tongue (*i.e.*, single-papilla-scale via the NTR) with solid chocolates or a hard palate and a contact of full-tongue with palate (*i.e.* tongue-scale via the rheo–tribo setup using biomimetic tongue surfaces). Friction behaviors of four chocolate samples were investigated in three stages of mouth–chocolate interactions: (A) licking stage: initial perception of the chocolate by tongue where chocolate is a countersurface, (B) molten chocolate/initial mastication: chocolate has undergone a phase transition to molten state, and (C) bolus before swallowing: chocolates mixed with the saliva. (**1**) In the licking stage (referred to as GoC), chocolate slabs (C) were rubbed against a glass probe (G) under a normal force (*F*_N_) of 50 mN and an entrainment speed (*u*) range of 0.5–25 × 10^–3^ m s^–1^. GoC-S resembles the condition where there is a salivary film on the tongue, emulated with a droplet of a model saliva (S, 20 μL) on chocolate surfaces. (**2**) For the experiments at the single-papilla-scale in stages B and C, G and polydimethylsiloxane (P) surfaces were used with a layer of molten chocolate (PoG or GoP) or its mixtures with S (PoG-S or GoP-S). The contact pressure (*P*_max_) and the *u* range were 120 × 10^3^ Pa and 0.05–25 × 10^–3^ m s^–1^, respectively. The contact radii (*r*_contact_) in PoG/PoG-S and GoP/GoP-S were 21 × 10^–5^ and 44 × 10^–5^ m, respectively. (**3**) The *P*_max_, *u* range, and *r*_contact_ at the tongue-scale were set to 45 × 10^3^ Pa, 0.05–50 × 10^–3^ m s^–1^, and 28 × 10^–5^ m, respectively. A schematic illustration of the bespoke tribo-setup equipped with a fluorescence microscope is shown at the bottom left of the figure, which was exploited to decipher the mechanisms of triboflow of the chocolate and chocolate-S samples at the single-papilla-scale. In PoG, the tribosweep action of cocoa particles that were confined at the contact interface was unveiled. In the PoG-S, oil-in-water (O/W) emulsion droplets surrounding the contact area with coalesced oil droplets at the contact interface along the fluid flow vortices were evidenced.

Schematic illustrations of tribological investigation of chocolate (C) and chocolate-S samples are presented in Figure 1. As shown in Figure 1, two versatile tribometers, namely, an NTR (Anton Paar, Switzerland) and a rheo–tribo setup (Kinexus Ultra+, Malvern Instruments, U.K.), were adapted to measure the frictional properties of the samples at the *single-papilla-scale* and *tongue-scale*, respectively.

The NTR was used to emulate a contact between a single-papilla (fungiform) and either a solid food (chocolate here) or the harder palate (resembled by glass surfaces) when there is a thin film of chocolate (or chocolate-S) existing between a papilla and the palate. Similarly, the rheo–tribo setup was employed to assess the frictional behavior of chocolate and chocolate-S samples in a full-tongue-scale configuration using the aforementioned tongue-mimic elastomers.^21^

A food substance undergoes several phases of dynamic processing across length and time scales in the mouth from the first instance that it is introduced to the mouth until it is swallowed, which can slightly differ depending on the nature of the food.^15,16^ For a solid chocolate, primarily three stages can be envisaged, which are referred to as *licking stage* (Figure 1A), *molten/initial mastication* (Figure 1B), and *bolus* (chocolate + saliva) *before swallowing* (Figure 1C).

In the licking stage, a chocolate is in a solid state and is directly in contact with the tongue papillae or saliva-wetted papillae (salivary pellicle on the surface of the tongue) where secreted salivary fluid interposes between the solid chocolate and the oral surfaces. This stage is simulated via a direct tribocontact of the G probe against solid chocolate slabs (C) in the absence or presence of 20 μL of S (37 °C) and hence is referred to as GoC or GoC-S, respectively (Figure 1(**1**)). In the *initial mastication* stage, the chocolate is converted to a molten state with no or very limited amount of mixing with the secreted saliva. Therefore, we carried out frictional measurements on molten chocolates with no addition of S to imitate this stage (Figure 1(**2** and **3**)). The *bolus before swallowing* resembles a condition such that the oral processing of a chocolate is complete and the mixture of the chocolate and secreted saliva is ready for swallowing. Here, chocolate samples were mixed with S (1:1 wt %) to mimic this stage (Figure 1(**2** and **3**)).

##### 2.2.5.1 Licking Stage (Figure 1A)

At first, 90% of the chocolate was tribosheared under different *F*_N_ values of 1, 10, and 50 mN (individual tests for each *F*_N_) at *u* = 0.5 × 10^–3^ m s^–1^ to evaluate the response of the frictional forces (*F*_t_) against the normal force and to obtain μ–linear position (ε) cycles. The *F*_t_ was spatially resolved by capacitive sensors when rubbed against the counterbody, which was mounted on a reciprocating piezoelectric actuator. A linear correlation between *F*_t_ and *F*_N_ was observed (Figure S4), which appears to be in agreement with the Bowden and Tabor theory^29,30^ (more information in Section S7). *F*_t_ values were recorded over a stroke amplitude of 2 mm in a reciprocating mode. The *F*_t_ values were logged with a data acquisition rate of 400 Hz between ε = ±800 μm and were used to obtain single coefficient of friction (μ) values for each sliding cycle. The *F*_N_ values of 10 and 50 mN were selected to be the same *F*_N_ values for the initial mastication and bolus before swallowing stages, assuming that similar forces are applied across all stages of oral processing (further details in Section S9).

Lastly, the influence of *u* on the frictional behavior of chocolates (70–99% cocoa) in GoC and GoC-S configurations was evaluated under a *F*_N_ = 50 mN over a *u* range of 0.5–25 × 10^–3^ m s^–1^ at three separate *u* values (0.5, 5, and 25 × 10^–3^ m s^–1^) (Figure 1(**1**)). Each measurement consisted of 50 reciprocating sliding cycles and were conducted on separate and fresh chocolate surfaces. The obtained μ values were independent of sliding direction and therefore, the μ readings from an individual cycle were averaged followed by the second averaging over 50 cycles to obtain a single value at each speed.

##### 2.2.5.2 Molten/Initial Mastication Stage (Figure 1B)

The experiments at the single-papilla-scale were performed in PoG (*i.e.*, the P probe on a G disc) configuration to simulate the contact of the soft tongue against the harder palate and GoP (*i.e.*, the G probe on a P disc) configuration to assess the influence of surface chemistry of the body providing the converging contact wedge,^11^ while the combination of contact-bodies stayed the same (Figure 1(**2**)). The probe was embedded into a holder, which is designed to be mounted into a quad beam force cantilever. A piezoactuator sensor tracks the movement (penetration or elevation) of the indenter in *Z* direction (*P*_d_). Molten chocolates (200 μL, 37 °C) were pipetted between the contact bodies and a sliding tribocontact started with *u* decreasing from 25 × 10^–3^ to 0.5 × 10^–3^ m s^–1^ (Figure 1(**2**)). The *u* range in this study covers the relative speed range between human tongue and palate (*i.e.*, 2–25 × 10^–3^ m s^–1^).^31^ The *F*_N_ for PoG and GoP configurations was set to 10 and 50 mN, respectively, to achieve the same Hertzian *P*_max_ of 120 kPa (eq 2). A *P*_max_ = 120 kPa is comparable to the contact pressures used in the literature, despite being slightly higher than the values reported for the real contact between the tongue and the palate (*i.e.*, 10–30 kPa^31,32^). The *r*_contact_ in the PoG configuration was estimated at 21 × 10^–5^ m using eq 3 (Figure 1(**2**)). The comparison between the PoG and GoP configurations was carried out at the same *u* values, and similar *r*/*r*_contact_ (GoP: 14.1 and PoG: 14.8) and theoretical *h*_min_ values (shown in Figure S5).

The experimental details at the tongue-scale are described in our previous work.^23^ Briefly, the positive replica obtained from the master mold was cut to 2 × 2 cm and attached to the plate of a rheometer (Figure 1) at a position where the centerline of the tongue-mimic sample was distanced 1.5 cm from the center point of the rheometer top plate. The tongue-mimic sample was compressed against the bottom plate of the rheometer at *F*_N_ = 1 N (Figure 1(**3**)). The molten chocolates were contained on top of the bottom plate (controlled at 37 °C) using a custom-designed pot made of an UV-curable polymer. The estimated *P*_max_ for the contact of a single fungiform papilla on the tongue-mimic elastomer and the bottom plate was 45 kPa, which is comparable to the values reported for the real tongue–palate contact in mouth.^32^ As soon as the load was stabilized, a unidirectional rotary tribocontact was propelled at an angular velocity (ω) ranging from ∼6.7 × 10^–3^ to 6.8 s^–1^ to obtain *u* values between ∼5 × 10^–5^ and 5 × 10^–2^ m s^–1^ (Figure 1(**3**)). Following at least a complete 2π rad rotation at each *u*, torque (τ) was recorded and eq 2 was used to calculate the corresponding μ
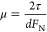
7where *d* is the diameter of the top plate (0.05 m). The gap size was tracked in a force-controlled mode to obtain the *P*_d_ data. The *r*_contact_ (eq 3) for the contact between a single-papilla and the bottom plate was estimated at 28 × 10^–5^ m, which is comparable to that for the PoG configuration at the single-papilla-scale (21 × 10^–5^ m) (Figure 1(**2** and **3**)). Since the *P*_max_ (eq 2) at the tongue-scale was close to the contact pressures reported for the real tongue–palate contact (*i.e.*, 10–30 kPa^31,32^), we decided to perform the compassion between the single-papilla-scale and the tongue-scale with a match-up of *r*_contact_ rather than *P*_max_. This was particularly considered based on our previous study, showing the significant influence of contact area on oral tribological results.^23^

##### 2.2.5.3 Bolus before Swallowing Stage (Figure 1C)

The experimental procedure, parameters, and apparatus in this stage were identical to the above (*i.e.*, initial mastication stage), while the chocolate-S samples (C:S = 1:1 w/w ratio) were used for this stage. The experiments at the single-papilla-scale on chocolate-S samples with the P probe against the flat G disc are abbreviated to PoG-S.

The G, P, and tongue-mimic surfaces were thoroughly cleaned between each experiment to eliminate traces of surface contamination. This included sonication steps of 10–15 min in sodium dodecyl sulfate (2 wt % in DI water), IPA, and DI water.

#### 2.2.6 *In Situ* Tribomicroscopy Setup

An in-house custom-built tribomicroscopy setup was exploited to reveal mechanisms governing the flow of chocolate (and chocolate-S) in an oral tribological context at single-papilla-scale in two stages of initial mastication (Figure 1B) and bolus before swallowing (Figure 1C). The setup shown in Figure 1 incorporates an electromagnetic reciprocating stage with embedded flat G surfaces. The setup is equipped with a fluorescence digital microscope (Dino-Lite Digital Microscope, AM4115T-GRFBY, The Netherlands), which emits fluorescent light at wavelengths 465 and 580 nm with magnification capabilities (20–220×) and dual-band emission filters of 505–540 and 610–720 nm. The microscope, located beneath the flat G, emits florescence through the G into the contact interface between the P probe and the G counterbody and visualizes the contact interface through the reflected florescent light captured by integrated detectors. Consistent with the PoG/PoG-S measurements via the NTR, a *F*_N_ = 10 × 10^–3^ N was applied delivering a *P*_max_ of 120 kPa and the tribocontact proceeded at 10^–3^ m s^–1^ and 37 °C. The 90% and 90%-S were selected as the representative samples. Tribomicroscopy was carried out for the stained and nonstained 90% and 90%-S samples. The results for nonstained 90% and stained 90%-S are presented due to their stronger contrasts under fluorescent light. Similar to confocal microscopy, a solution of Nile red (1 g L^–1^) was added to 90%-S at a final concentration of 0.02 g L^–1^ immediately before the tribomicroscopy.

#### 2.2.7 Chemical Analysis

A PerkinElmer Spotlight 400 attenuated total reflection-Fourier transform infrared (ATR-FTIR) spectrometer was used to assess the surface of the G probe after the tribocontact in GoC configuration.^26,33^ ATR-FTIR spectra were obtained over a wavelength range of 650–4000 cm^–1^. Reference spectra were collected from the surfaces of cleaned G and pristine 90% chocolate in the solid state for cross-comparison. The identified peaks were assigned using the spectra from the Sigma library of FTIR.

#### 2.2.8 Statistical Analyses

All measurements were carried out at least three times on triplicate samples prepared on separate days immediately before measurements and are reported as the mean and standard deviation (*n* = 3 × 3) unless otherwise specified. Statistical analyses were performed using one-way analysis of variance (ANOVA) using Tukey test and the significant difference between samples was considered when *p* < 0.05.

## 3 Results and Discussion

### 3.1 Size of Solid Particles in Chocolates Was Similar

**Figure 2:**
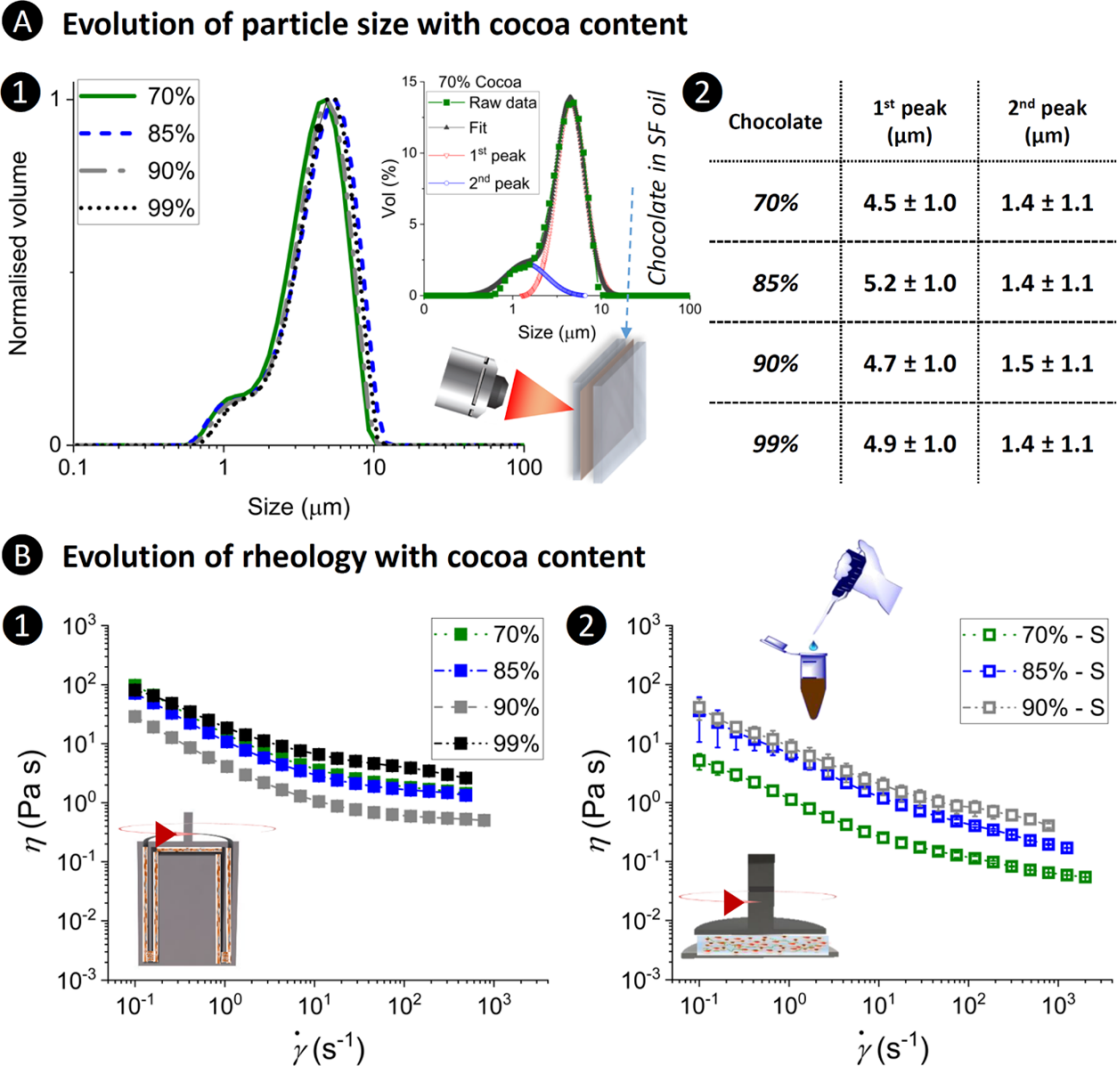
Evolution of size and flow behavior with cocoa content. (A) Particle size distribution of diluted chocolate samples in sunflower (SF) oil obtained via static light scattering using a Mastersizer and (B) flow behavior of (1) chocolate and (2) chocolate-S mixtures at a ratio of 1:1 wt %. (A1) Superimposed graphs (normalized volume–size) of chocolate samples showing negligible differences in particle size distribution of the chocolate samples irrespective of cocoa content. The graphs were fitted using the Gaussian function to derive two distinctive peaks. The resultant mean particle sizes are listed in panel (A2), showing two distinct particle sizes ranging 4.5–5.2 ± 1.0 and 1.4–1.5 ± 1.1 μm. (B1) Apparent viscosity (η)–shear rate (γ̇) graphs obtained using a double-gap geometry, showing shear-thinning behavior of chocolate samples reaching a high-shear rate plateau at the highest shear rate (η_∞_). (B2) Chocolate-S mixtures (except for 99% chocolate) showing pseudoplastic behavior across the measured γ̇.

The size distribution of solid particles in chocolates is an important parameter to determine whether the particles entrain into the tribocontact gap and hence affect the tribological performance. This is likely when the size of cocoa particles is considerably smaller than the size of the gap.^8,34^ For a true comparison, the volume density for each chocolate was normalized to the peak maximum of the more pronounced peak (*i.e.*, the peak emerged at a larger size, Figure 2A1). All of the chocolate samples in this study showed a bimodal PSD. The two Gaussian peaks obtained for each chocolate appeared within a range of 0.6–10 μm (Figure 2A1), showing no significant size difference between the chocolate samples for a given peak (*p* > 0.05).

The obtained PSD is similar to the profiles observed for dark chocolates with a cocoa particle size (*D*_90_) of ∼18 μm.^7^ The most prominent peaks for each chocolate showed maxima at 4.5–5.2 ± 1.0 μm (Figure 2A2), which was close to the mean particle size (*D*[4,3]) of the corresponding chocolate (see Table S1). The specific surface area of the particles (shown in Table S1) varied between 1.091 and 1.225 m^2^ g^–1^ in agreement with previous reports.^7^ Thus, this suggests that tribological differences (if any) between the chocolates cannot be attributed to the particle size.

### 3.2 Flow Behavior Changed on Mixing with Saliva

Apparent viscosity is a hallmark feature to determine the hydrodynamic forces that generate a lift force to separate the contacting bodies and consequently enable entrainment of the particles in between the tribopair. All of the chocolate samples showed shear-thinning behavior (Figure 2B), despite the Newtonian behavior of cocoa butter.^8^ Only the chocolate samples reached a high-shear rate plateau (η_∞_). A nonmonotonic relationship was observed between η of the chocolates and either their fat content or cocoa particle concentration (Figure 2B1). The 70 and 85% chocolates showed an almost identical η – γ̇ behavior showing intermediate η values between the most viscous sample (99%) and the thinnest sample (90%). This might be attributed to the various processing conditions for chocolates (conching, tempering, and temperature) and different volume fractions of cocoa, fat, and lecithin (emulsifier) concentrations in the chocolates, influencing the interactions between the particles, cocoa butter, emulsifier, and the protein ingredients (Tables 1 and S1).^7,35^

The chocolate-S mixtures also demonstrated a pseudoplastic behavior across the whole γ̇ window measured in this study (Figure 2B2). Unlike for the chocolates, the η – γ̇ graphs for the chocolate-S mixtures showed a direct correlation between η and the fat/cocoa solid content. The aggregation/jamming of the particles is expected to increase at higher concentrations of cocoa particles and hence is a contributing factor to the higher η values of chocolate-S samples containing higher cocoa contents.^35^ In fact, the decreased particle crowding in the chocolate-S mixtures was expected to reduce interactions between particles due to dilution as compared to the parent samples (without saliva) and such dilution may reduce η values.^9^ This was observed to be the case for 70%-S and 85%-S samples (Figure 2B2 and B1). However, the addition of S enhanced η values of 90% chocolate. The reason for this is not entirely clear and might have arisen from the capillary forces bridging solid particles in the 90%-S sample.

**Figure 3:**
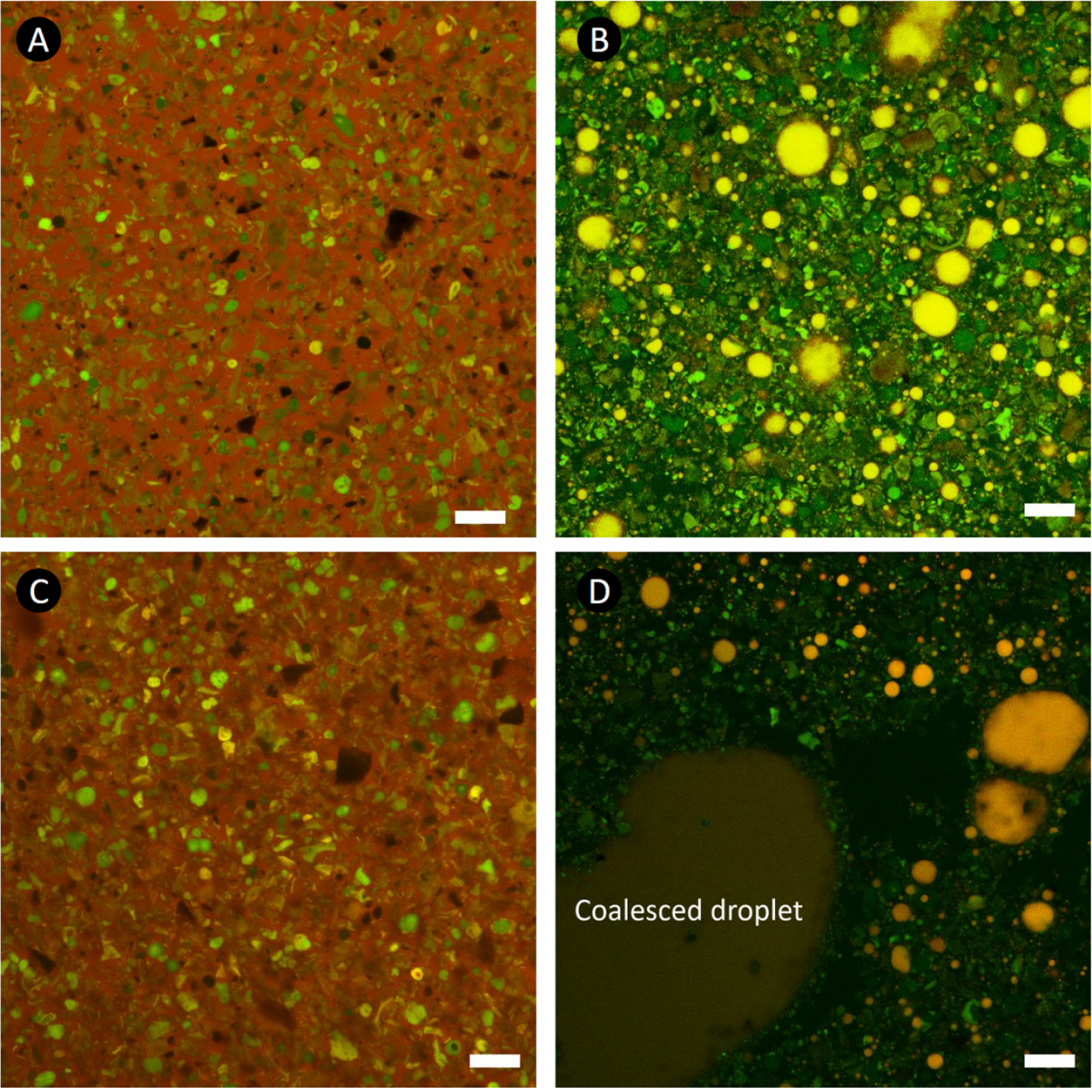
Microstructural evolution of chocolates on triboshearing. (A, B) Confocal images of pristine 90% and 90%-S samples, respectively. (C, D) Post-triboshear structure of pristine 90% and 90%-S samples, respectively. For the images of 90% chocolate, the green particles, black angular voids, and red continuous phase show solid cocoa particles, sugar particles, and fat matrix, respectively. The orange-red and green contrasts represent the fat droplets and the solid cocoa particles dispersed in the black aqueous phase, respectively. The white scale bars in the images represent 20 μm.

On addition of S, the chocolate samples were converted into an oil-in-water (O/W) emulsion system (see the microstructural evolution from Figures 3A–B and S3) with their droplets most likely coated by lecithin and cocoa particles. This was apparent as the O/W emulsion containing orange/red fat droplets (Figure 3B), which is in close agreement with previous reports on expectorated boli and in-lab mixtures of saliva and chocolates.^8,9^

The change to an O/W emulsion brings about new interactions (cocoa particle–cocoa butter,^36^ cocoa–cocoa in S, salivary mucin–cocoa particle, salivary mucin–lecithin, etc.) or mucin-induced depletion flocculation^14,25,37^ (often manifested in enhancement of viscosity), which appeared to dominate the rheological behavior of the chocolate-S mixtures. Consequently, the extended shear-thinning behavior of the chocolate-S samples until higher γ̇ values (Figure 2B2) can be attributed to the higher shear stresses required to break S-induced aggregates of cocoa particles/fat droplets and to disturb the S-triggered interactions.^24^ The tribosheared microstructural changes of the chocolates (Figure 3C,D) are discussed later.

### 3.3 Stage/Scale-Dependent Tribological Behavior

For the tribological experiments, we hypothesized that the oral processing of chocolates starts with a (1) licking process, experiences a phase change to a (2) molten-state, and eventually evolves to (3) saliva-mixed state.

#### 3.3.1 Licking Stage

**Figure 4:**
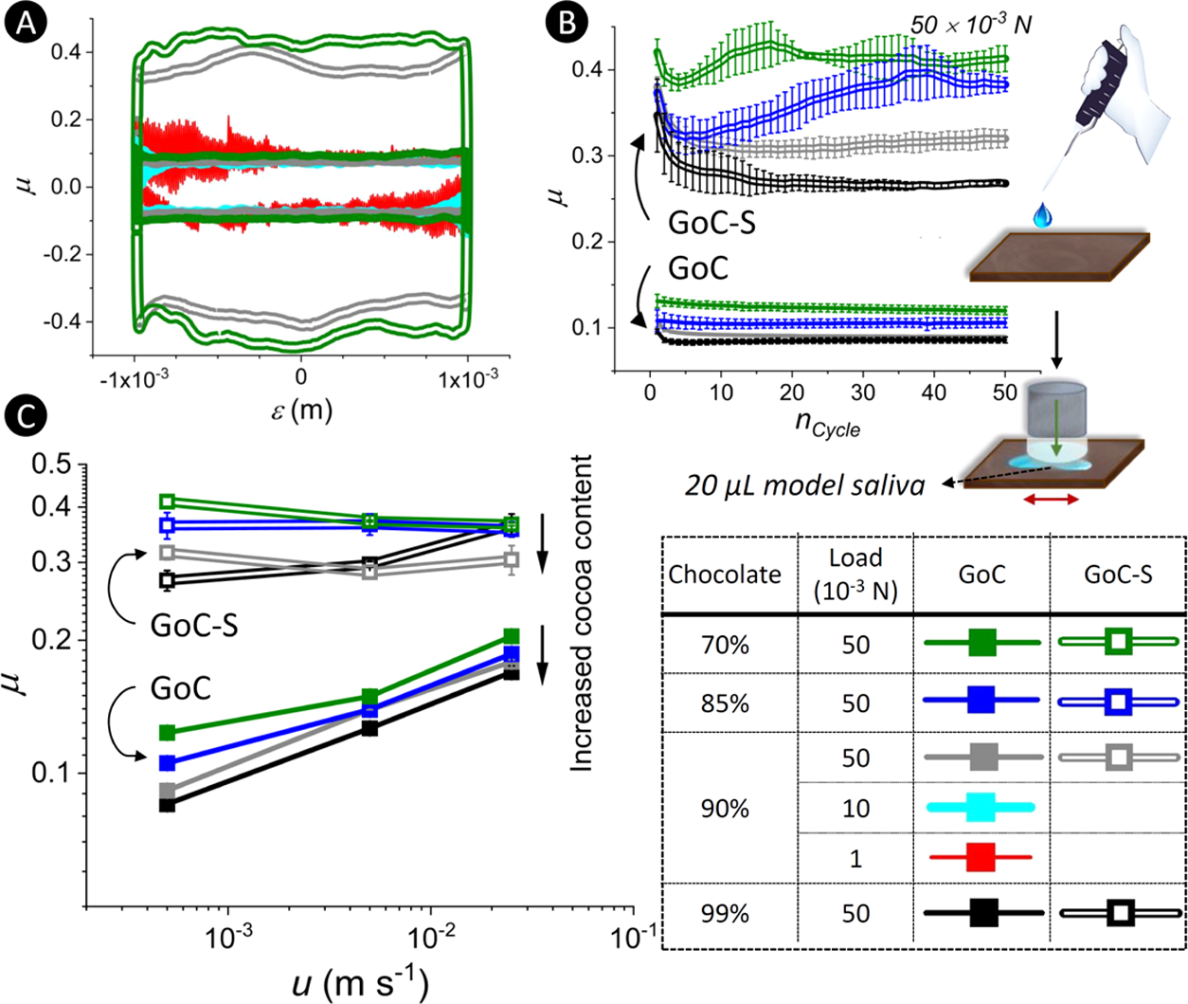
Frictional behavior of nonmolten solid chocolate at the single-papilla-scale simulating the licking stage. (A) Reciprocal friction coefficient (μ) results showing a μ–linear position (ε) cycle for the 90% chocolate rubbed against a G probe (GoC) under different normal loads (1, 10, and 50 × 10^–3^ N) along with GoC results for the 70% chocolate under 50 × 10^–3^ N and S-wetted (20 μL, 37 °C) chocolate slabs (GoC-S) under 50 × 10^–3^ N. (B) μ graphs in the GoC (-S) configuration across 50 cycles of the sliding-contact under 50 × 10^–3^ N showing efficient solid lubricity of the chocolates in the lack of a salivary film. The image shows the procedure used where chocolate and G counterbodies had 20 μL of saliva as a lubricant. C. presents the μ–entrainment speed (*u*) graphs showing an inverse correlation between the cocoa content and the μ values across the measured *u* for both GoC and GoC-S measurements.

The tribological results for GoC/GoC-S configuration are presented in Figure 4. Figure 4A shows a typical reciprocating cycle of μ – ε at *u* = 0.5 × 10^–3^ m s^–1^. The μ – ε varied to a higher degree in the GoC-S configuration, which suggests an occurrence of tribo-induced interfacial interactions between S and the chocolates (Figure 4A).

The μ values as a function of number of tribocontact cycles (*n*_Cycle_) are presented in Figure 4B, showing a drop in the μ within the initial five cycles. The *P*_d_ results evidenced penetration of the G into the chocolates during sliding (shown in Figure S6), which together with the drop in the μ, suggests plastic deformations of the chocolate and S-wetted chocolate surfaces and potentially a drop in the contact pressure as the sliding progressed. This behavior is a well-known phenomenon typical in tribology of hard surfaces,^38^ where plastic deformations of the surface/subsurface layers occur, often referred to as the running-in effect. Following the drop in the μ, the μ – *n*_Cycle_ curves for the chocolate samples (GoC) levelled off, indicating a steady-state frictional process, while the GoC-S curves showed dynamic processes (Figure 4B).

The μ in GoC and GoC-S at *u* values of 0.5, 5, and 25 × 10^–3^ m s^–1^ are shown in Figure 4C, exhibiting an inverse correlation between the cocoa content and the μ. For a given chocolate, μ values (Figure 4C) in GoC-S were significantly higher (by 50–300% depending on the *u*) than those in GoC across all measured *u*. A *u*-dependent friction behavior was observed in GoC, as opposed to the relatively unchanged μ – u behavior in GoC-S, which can be perceived through disparate lubrication mechanisms in each configuration.

The GoC essentially can be explained through the “*solid-lubrication*” concept. The solid lubricants such as graphite, diamond-like carbon-coatings, MoS_2_, etc. are commonly used for demanding applications where a fluid lubricant cannot be used (*i.e.*, in high temperature or vacuum applications).^39^ Often a transfer film (*i.e.*, detached layers of a solid lubricant, which are deposited/bonded onto the counterbody) is formed on the counterbody facilitating an easy-slip plane that moderates the frictional forces.^39^ A similar mechanism governs the tribocontact in GoC; that is the transfer of a continuous layer of cocoa butter (fat layer evidenced via ATR-FTIR—Figure S7) from worn-out chocolate to the G surface and effectively leading to a triboshear between fat layers. Therefore, it can be envisaged that chocolates with higher fat contents show lower μ values (Figure 4 and Table 1). Solid lubricants often show a speed-dependent performance due to the fact that heat dissipation and kinetics of transfer film formation and removal are influenced by the sliding speed.^39^ Tribological processes are generally nonadiabatic and frictional forces generate significant local heating at the contact interface, which often intensifies at higher sliding speeds. Therefore, it can be implied that a greater frictional heat dissipation at higher speeds partly melts the fat transfer film, hence accelerating the squeeze-out dynamics of the transfer film and increasing the μ (Figure 4C).

The chocolates in GoC-S experienced a different lubrication mechanism, that is, an aqueous film flooding the contact, probably facilitated by the hydrophilic nature of the G. The interposition of an aqueous film between the contact bodies (*i.e.*, chocolate and G) is expected to hinder the formation of easy-slip fat layers. It is clear that a thin film of S (with poor surface lubricity^11,25^) cannot provide the desirable solid-fat lubricity of the transfer film in GoC,^11^ which justifies a nearly three–four fold increase in μ values in GoC-S. One might argue why the friction coefficient increases dramatically on adding S, despite S being well-acknowledged as a high-performance biolubricant.^2^ It is important to note that saliva acts as a boundary lubricant only when it is unstimulated and markedly loses its lubrication performance upon stimulation, such as oral processing, which is the case here.^25^ Noteworthy, PGM has been used in this study to prepare the model saliva, which in fact has poor boundary lubrication performance,^25^ but emulates the increased friction as might be observed in presence of stimulated saliva upon oral processing. In other words, such an increase in friction on moving from the saliva-poor regime to the saliva-rich regime is highly likely in real biological contexts.

#### 3.3.2 Initial Mastication/Molten Stage

**Figure 5:**
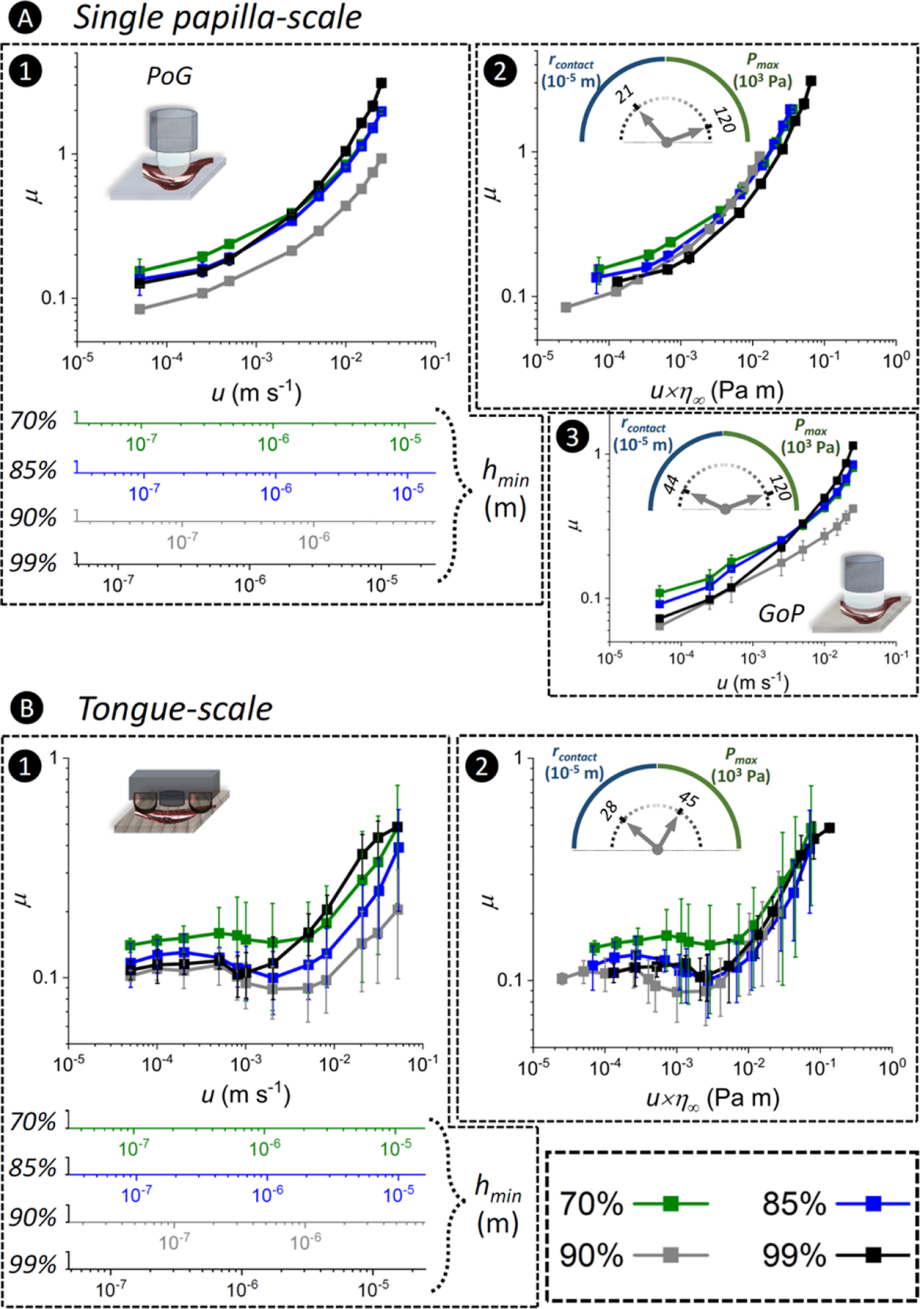
Frictional behavior of molten chocolates in the absence of model saliva during the initial mastication stage. Friction results for measurements in the *initial mastication* stage at the (A) single-papilla-scale and (B) tongue-scale. The friction coefficients (μ) are shown as functions of (A1, B1) the entrainment speed (*u*) and (A2, B2) the product of *u* multiplied by the high-shear rate viscosity (*u* × η_∞_). (A1, A3) μ – *u* graphs in PoG and GoP configurations, respectively, at the same value of *P*_max_.

The μ results in the initial mastication stage at the single-papilla-scale and the tongue-scale are shown in Figure 5A and B, respectively. Using eq 6 and the obtained η_∞_ values (Figure 2B1), the derived *h*_min_ values (across the measured *u*) at the interface of P probe and the individual fungiforms on the tongue-mimic surfaces are presented below the *x*-axis in Figure 5A1 and B1, respectively.

The log (*μ*)–log (*u*) graphs at the single-papilla-scale (Figure 5A1) showed an inverse correlation between the fat content and the μ values, indicating the pronounced influence of cocoa butter. The log (μ) increased in a nonlinear manner as a function of log (*u*). This nonlinear behavior does not appear to agree with the classical elasto-hydrodynamic lubrication (EHL) theories^11^ and probably resulted from high solid particle contents of the chocolates, which is not a common consideration in the classical tribological investigations.

In this study, the confinement of cocoa solids at the contact interface was evidenced (Image S1), which to our knowledge, is the first study that visualized cocoa particle confinement. In classical tribology of hard surfaces, solid wear particles can be trapped between contacting surfaces but they tend to indent the surfaces and induce abrasive wear (*i.e.*, a plastic deformation mechanism). Chocolate particles have been shown to indent soft plastic surfaces (polytetrafluoroethylene) and to embed into the materials^18^ influencing frictional behavior; however, such indentation into elastic materials or a real tongue tissue remains disputable.

According to eq 6, the estimated *h*_min_ increases at higher *u and η values* (*h*_min_ ∝ η_∞_^0.68^), which is reflected in Figure 5A1 and B1 (*e.g.*, 99% chocolate has highest *h*_min_). The estimated *h*_min_ values (Figure 5A1) appeared to be smaller than the average particle sizes of 70 and 85, 90, and 99% chocolates (Figure 2A, ∼5 μm) until *u* values of ∼2 × 10^–2^ (*i.e.*, almost the end of the *u* range), 5 × 10^–2^, and 5 × 10^–3^ m s^–1^, respectively. This suggests that under the defined conditions in this study, the solid particles cannot entrain into or out of the contact interfaces^34^ except for 99%. On the other hand, the confinement of particles at the contact interface suggests that the separation between the contacting surfaces will be larger than the estimated *h*_min_. The particle confinement is probably accompanied by the elastic deformation of P surfaces. The particle confinement indicates that the lubrication behavior of chocolates cannot be explained solely by theoretical estimation of *h*_min_. In other words, nonconformity of the μ behavior with the classic lubrication theories (*i.e.*, nonlinear log(μ) – log(*u*) mentioned above) was observed for these materials. This was also manifested in a work by Rodrigues et al.^9^ where the μ for dark chocolates plateaued where hydrodynamic forces could not generate sufficient contact gap larger than the size of cocoa particles.

*P*_d_ observations (Figure S8A) showed a gradual drop (at *u* < 2 × 10^–2^ m s^–1^) followed by a plummet as *u* increased, indicating smaller contact gaps at higher *u* values. Considering the particle confinement and *P*_d_ observations, we postulate that the lubrication of surfaces in the single-papilla-scale is governed by a bridging effect of cocoa butter in between the cocoa particles, which was impaired at higher *u* values. This explains the lower μ values of the chocolates with higher fat contents (Figure 5A1). Consequently, the interplay between removal and replenishment of the bridging cocoa butter plays a pivotal role. Similar to the GoC results (licking stage, Figure 4), it can be envisaged that the bridging cocoa butter was depleted or failed to replenish the contact interface at higher *u* values. Therefore, the probability of a direct contact between the confined solid particles and the contact surfaces increased, resulting in higher μ values^11,15^ (*i.e.*, impaired bridging effect of cocoa butter). This behavior persists until the gap is sufficiently large to accommodate the entrainment of cocoa particles. The sharp increase in the μ of 99% chocolate (showing the largest *h*_min_ values) at *u* > 2 × 10^–3^ m s^–1^ (Figure 5A1) corresponds to *h*_min_ > 2 μm, suggesting a transition from the bridging cocoa-butter mechanism alone to the entrainment of solid particles, which has been shown to increase the μ of dark chocolates.^8^ Therefore, an interplay between the fat content, solid particles, and viscous forces determines the friction properties of dark chocolates.

The μ – η_∞_ × *u* graph at the single-papilla-scale is shown in Figure 5A2, representing a μ – *u* relationship where the influence of η is minimized.^11,28^ For Newtonian fluids with no pronounced interfacial interactions (*e.g.*, surface adsorption or particle inclusion) or a viscous force-induced surface separation effect,^40^ the frictional forces at the contact surfaces are expected to be independent of the viscosity of the lubricating fluids.^11^ As can be seen in Figure 5A2, the μ – η_∞_ × *u* curves did not completely collapse into a single curve, which probably stemmed from the particle confinement or entrainment.

In the GoP configuration, lower μ values with increased fat contents and the particle entrainment for 99% chocolate were observed (Figure 5A3) similar to the PoG configuration, albeit the μ values were different for a given chocolate.

The lubrication mechanism for the tongue-scale setup has been discussed in detail in our previous works.^21−23^ Briefly, the tongue-scale testing uses a flat-on-flat configuration, exploiting a fluid reservoir in between papillae to feed the contact interface.

The converging contact wedge at the papilla level can generate hydrodynamic lift forces (*i.e.*, fluid pressurization), leading to a *u*-dependent lubricant film formation at the interface of individual fungiforms.^23^ The curved nature of the papillae sets it apart from the classical definition of “peak” in tribology of rough surfaces. A similar curved-hill topography was shown to generate a micro-hydrodynamic wedge action, which reduced the μ in the mixed lubrication regime depending on the number and radii of summits,^41^ albeit in hard metallic contacts and lower surface roughness values.

At the tongue-scale, a monotonic friction behavior was observed for all chocolates at approximately *u* < 5 × 10^–4^ m s^–1^, followed by a slight drop within a small *u* range (5 × 10^–4^ m s^–1^ < *u* < 5 × 10^–3^ m s^–1^ for 70, 85, and 90% chocolates) and later a linear increase in log (μ) – log (*u*) at *u* > 5 × 10^–3^ m s^–1^ (Figure 5B1). The *P*_d_ curve (Figure S8B) remained relatively unchanged at the monotonic μ regime and later increased gradually and intensely where the slight drop and the linear increase in μ were observed (Figure 5B1), respectively. This suggests a steady-state contact between fungiform papillae on the tongue-mimic and the counterbody within the monotonic μ regime. In the monotonic regime, the chocolates with higher fat contents showed lower μ values (μ: 90% < 99% < 85% < 70%), suggesting a probable fat-driven lubricity mechanism (a layer of cocoa butter at the interface). This corroborates the bridging effect of cocoa butter, which facilitated lubricity and reduced the occurrence of direct contact between the solid particles and the surfaces in the PoG configuration. The slight drop in the μ and the coinciding increase in *P*_d_ imply a micro-hydrodynamic effect, meaning that the contact load was partially supported by a continuous thin film of cocoa butter. The marked increase in the μ (linear log (μ) – log (*u*)) and the coinciding increase in *P*_d_ at higher *u* values indicate a hydrodynamic lubrication regime, which provided a full separation of surfaces by a full film of cocoa butter or chocolates. The transition to the EHL regime was subtle at the tongue-scale measurements compared to what is often observed in the classical EHL theories. This may be attributed to the topography of the tongue-mimic, which is orders of magnitude rougher than the typical engineering surfaces.

The μ – η_∞_ × *u* graphs at the tongue-scale (Figure 5B2) showed a fat-content-dependent μ behavior in the monotonic and hypothesized micro-hydrodynamic regimes, which agrees with the abovementioned cocoa-butter bridging effect when surfaces are in close proximity. The μ – η_∞_ × *u* graphs collapsed into a single curve at η_∞_ × *u* > 10^–2^ Pa m bearing out the hydrodynamic lubrication regime (*i.e.*, a regime where viscosity of the full film determines the lubrication behavior). A higher fat content appeared to shift the onset of the micro-hydrodynamic effect to lower η_∞_ × *u* values (*i.e.*, accelerated the μ drop). In other words, higher fat levels delayed the occurrence of direct contact between the solid particles and the surfaces until lower η_∞_ × *u* values were reached.

Two similarities between the tribological behavior at the tongue-scale and the single-papilla-scale were the sharp increase in μ of 99% (suggesting entrainment of the solid particles) and the inverse correlation between the fat content and the μ. However, disparate lubrication mechanisms appeared to dominate the friction behavior, essentially due to the topography of tongue-mimic surfaces. It is worth noting that as a result of a meticulous selection of tribocontact parameters (*F*_N_, *r*′, and *E*′ of PoG and tongue-scale), the theoretical *h*_min_ values at a given *u* for a given chocolate were similar at both scales, making comparisons more robust.

#### 3.3.3 Bolus before the Swallowing Stage

**Figure 6:**
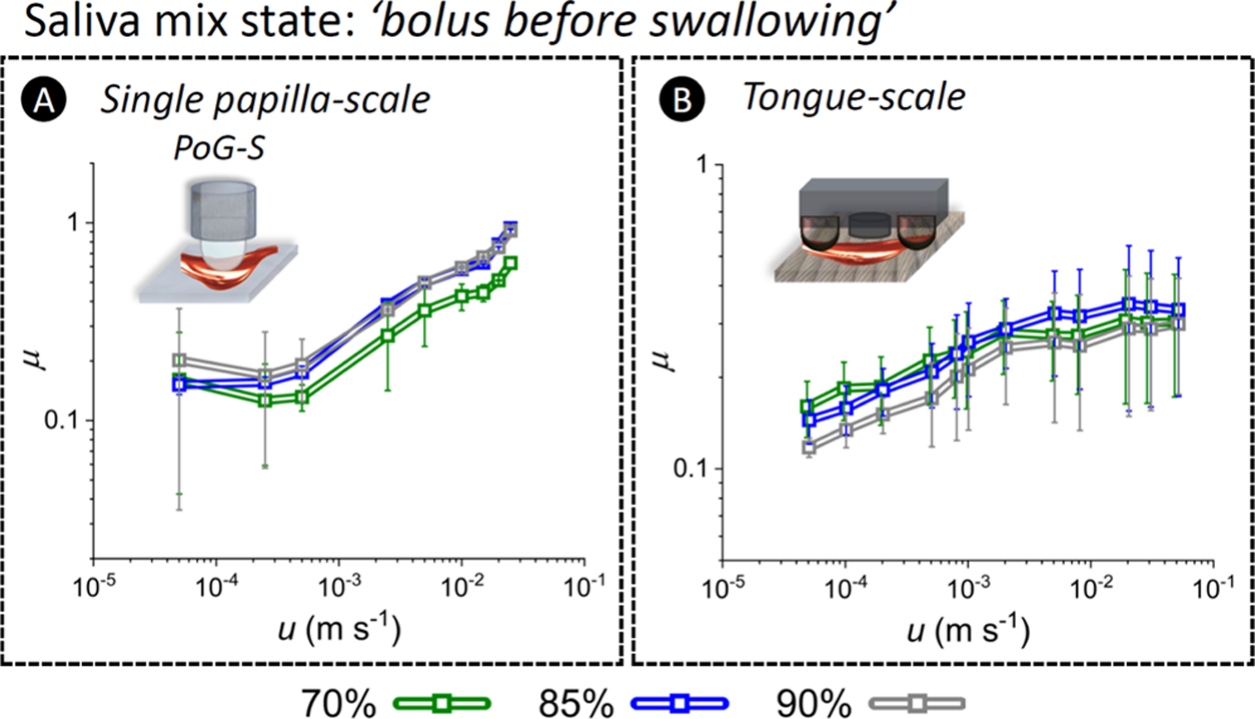
Frictional behavior of chocolate-S bolus. The coefficient of friction (μ) as a function of the entrainment speed (*u*) in *bolus before the swallowing* stage at the (A) single-papilla-scale and (B) tongue-scale. The dissimilarities in the μ behavior can be attributed to the disparate lubrication mechanisms across scales due to the differences in surface topography, contact configuration, and wettability of surfaces. The fluid film at the contact interface became thinner at the single-papilla-scale, while it grew thinker at the tongue-scale.

Unlike the chocolate samples that did not experience a noticeable microstructural change following the tribocontact (Figures 3A,C and S3), a substantially tribocontact-induced structural transformation was observed for the chocolate-S samples. Extremely large coalesced oil droplets in the order of 200 μm (1 mm in some cases) emerged (Figures 3B,D and S3). The coalescence of oil droplets as a result of tribocontact has been postulated in the literature,^8,9^ but our finding presents the first visualized *in situ* evidence for such a structural change. This dynamic change can be suggested as the underlying factor in high deviations of μ values observed in Figure 6. Further, Figures 3D and S3 showed a rearrangement of autofluorescent green cocoa solid particles to form a corona shell surrounding the coalesced droplets. Cocoa particles have been used as a Pickering stabilizer for emulsions of O/W,^36^ and it is interesting to observe that the tribocontact prompted such an effect, *i.e.*, cocoa particle-laden droplets.

Interactions of salivary mucin molecules with the contact surfaces (*i.e.*, adsorption, bonding, etc.) can influence the tribological measurements.^11,22,25^ However, the PGM used in this model saliva has poor surface effects,^24,25^ and hence its interfacial influence on the μ behavior is expected to be negligible in this study. The tribomicroscopy setup evidenced an oil-lubricated contact interface in the PoG configuration (Figure S9 and Video SV1), which can be attributed to the greater η (at least one order of magnitude) of the cocoa butter as compared to that of S.^8^ Our observations closely resemble previous reports suggesting coalescence of oil droplets at the contacting surfaces.^8,9^Figure S10 and Video SV2 show dynamic occurrences of coalescence of oil droplets into bigger droplets and split of large oil droplets into smaller droplets around the contact area where the shadow of flow vortices was more chaotic.

At the single-papilla-scale, the apparent μ behavior (Figure 6A) suggested a positive correlation between the cocoa solid content and the μ in contrast to the fat-content dependency in the initial mastication stage (Figure 5A). We also noted a correlation between the η and μ values of the mixtures. Our results did not suggest a confinement of solid particles for the chocolate-S samples (Figure S10 and Video SV2). Further, *P*_d_ values (Figure S8A) showed larger contact gaps at the lower *u* values. Therefore, the increase of μ as a function of increased *u* can be attributed to higher proportions of direct contacts between the contact bodies as the thickness of the coalesced oil layer reduced.

At the tongue-scale, it is debatable to affirm the dominant lubricating phase at the interface. This is due to the higher affinity of the tongue-mimic elastomer to aqueous phases compared to that of P and potential interactions between mucins from S and the tongue-mimic elastomer influencing the wettability of surfaces. Comparable μ values were observed for the 70%-S and 85%-S samples with slightly lower μ values for 90%-S across all *u*, which may suggest a fat content-dependent μ behavior (Figure 6B). The μ – *u* plot in Figure 6B showed a minor μ increase from 0.1 to 0.3, which are comparable values to the μ values observed for the monotonic regime in the initial mastication stage (Figure 5B1). This suggests the presence of a growing coalesced oil film at the interface, which appeared to corroborate the gap separation (*P*_d_) results showing larger *P*_d_ values at higher *u* values (Figure S8B).

**Figure 7:**
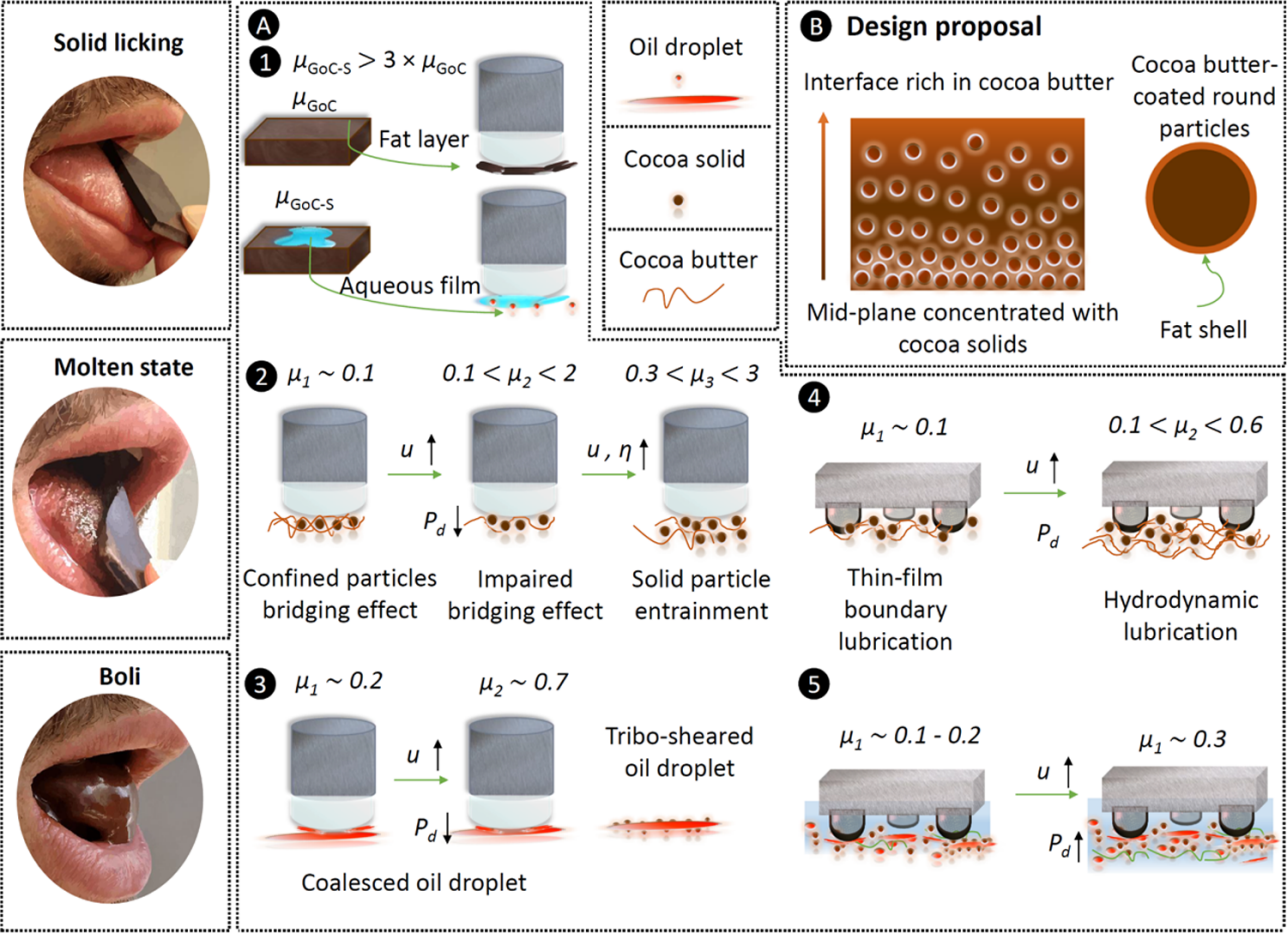
Summary of lubrication mechanisms of edible PCM across scale and proposed design of tribology-informed future fat-reduced dark chocolates. (A1) In the solid licking stage, a continuous fat layer accommodates the contact between solid chocolates and glass surfaces, bringing about coefficient of friction (μ) values of μ_GoC_ = 0.1–0.2. The existence of a salivary film on the surface of solid chocolates shifts the governing mechanism to aqueous lubrication, leading to around a threefold increase in μ (μ_GoC-S_). (A2 and A4) In molten/initial mastication stage, the bridging effect of cocoa butter in between confined cocoa particles at the contact interface provides μ values similar to μ_GoC_ at low entrainment speeds (*u*); μ_1_. As *u* increased, the cocoa-butter bridging impaired for the mesoscale and the chocolate film entrained into the contact at the macroscale, resulting in increased μ (μ_2_). At higher *u* values, solid cocoa particles entrain into the contact interface, especially for chocolates with relatively high viscosity (η) values, further increasing the μ (μ_3_). (A3 and A5) In the bolus before the swallowing stage, coalesced oil droplets at the interface lubricate the contact interface. (B) Tribologically informed design of dark chocolates with lower fat content but with superior lubricity is proposed: a gradient design with higher concentration of cocoa butter at the top surface and sufficient cocoa butter to bridge cocoa particles. The former provides the desired licking experience, and the later enhances the bridging effect and alleviates frictional stresses, hindering cocoa particles to come into direct contact with tongue surfaces.

Figure 7 presents an illustration of governing lubrication mechanisms across the stages (Figure 7A1–A3 or A4–A5) and scales of oral processing (Figure 7A2 and A4 or A3 and A5). Based on the findings, we propose a gradient design of future chocolates (Figure 7B), which may improve the oral sensory perception of dark chocolates even with lower fat contents. The novel methodology in this work can accurately distinguish solid lubricity of chocolates with marginal differences, in this case the cocoa content. We showed that in the licking stage, a fat content-dependent stage, the lubricating film evolves from a continuous fat layer to a rather aqueous salivary layer, the latter showing remarkably higher μ values (Figure 7A1, μ increased by around threefold). Therefore, our hypothesis is that if a layer high in fat saturates the surfaces of chocolates (Figure 7B), saliva may not be able to form an aqueous film or supress the continuous fat layer. Therefore, the lubrication through a fat layer remains dominant until next stages of mastication, and consequently, a better sensory perception may be anticipated.

In the molten state, the composition of the continuous lipid phase is shown to be the leading factor in lubrication performance. At the single-papilla-scale and the initial low-*u* region of the tongue-scale, our results evidenced the governing influence of cocoa butter, bridging the confined cocoa particles hindering a direct contact of the coarse cocoa particles with the papillae in mouth (Figure 7A2 and A4). The cocoa butter has been shown to reduce plastic viscosity (resistance to flow)^5^ and act as a “wetting fat” phase surrounding the particle surfaces.^5^ The birding effect was impaired as *u* increased, leading to higher μ values (Figure 7A2). Depending on the η of chocolates and *u*, a sufficiently large contact gap accommodates the entrainment of coarse cocoa particles and hence increases frictional forces (Figure 7A2), probably leading to a gritty or sandy perception of dark chocolates. Stiff and sharp-faceted particles often lower the perception threshold and show significantly higher μ values.^42^ We therefore, hypothesize that a layer of soft cocoa-butter-coating at the interface of cocoa particles can facilitate the cocoa-butter bridging and alleviate the friction-enhancing behavior of entraining/confined cocoa particles (Figure 7B). At the macroscale, the topography of the tongue-mimic supported entrainment of cocoa butter at high *u* values and hence a distinct hydrodynamic regime with a subtle EHL regime was observed (Figure 7A4). The high viscosities of the chocolates provided the hydrodynamic forces to separate the surfaces (Figure 7A4), which have shown to play a pivotal role in the perception of creaminess.^5^

At the later stage of oral processing where stimulated saliva makes solvation of the ground and molten chocolates, the triboshear prompted dynamic structural changes to the O/W emulsion and coalesced oil droplets emerged at the contact interface (Figure 7A3 and A5). We observed Pickering oil droplets coated by gritty and coarse cocoa particles, which might contribute to the perception of mouth-coating with dark chocolates. This is because the cocoa particles may hinder the oil coverage of oral epithelial surfaces which has been suggested as a pleasant mouth-feel obtained with chocolates.^5^ Therefore, as shown in Figure 7B, cocoa particles coated with a monolayer of cocoa butter may reduce such coarse mouth-coating feeling. In summary, we propose a multiscale tribology-informed smart design of chocolate in Figure 7B, with a “gradient” architecture having high fat at the surface and limited fat in the bulk phase containing fat-coated cocoa particles. Future work that combines such instrumental assessments reported in this work with sensory trials can evaluate whether or not the proposed gradient structure in Figure 7B offers just-right mouth-feel with significant calorie reduction.

## 4 Conclusions

Here, we present the first systematic investigation of the multiscale lubrication mechanism of an edible PCM containing solid particles (*i.e.*, chocolate) supported by relevant theoretical considerations when undergoing a phase change and mixing with a biolubricant (*i.e.*, saliva) during different stages of oral processing. The current setups used in this study, by far, provide the closest approximations to the real tongue–palate contact compared to previous works. At the tongue-scale, the fat content of dark chocolates appeared to be the most influential factor on the lubrication behavior across the stages of oral processing and parameters defined in this study. At the single-papilla-scale, the solid lubricity of the studied chocolates correlates with their molten-state lubricity; however, the correlation was disturbed in the presence of saliva. Using tribomicroscopy at the single-papilla-scale, we observed confinement of the cocoa particles at the contact interface for molten chocolates and, therefore, we argue that the classical lubrication theories cannot fully explain the tribological behavior of edible PCMs. The entrainment of solid particles into the contact can increase the friction coefficient, provided that the hydrodynamic forces are sufficient to facilitate the particle entrainment. Multiscale characterization methodologies from this work can be used as robust reference to decipher the often peculiar tribobehavior of PCM materials when they undergo phase transformation. Altogether, we hope that the knowledge is conceptually attractive to facilitate engineering of PCM and other metamaterials that are often subjected to tribological stresses.

## Supporting Information Available

Details of composition and particle size of chocolate samples; model saliva recipe; rationale behind using the ratio of 1:1 for chocolate-S samples; image of the tongue-mimic tribological setup; rheological performance of chocolates measured using various geometries; confocal micrographs of pristine and tribosheared chocolate samples; frictional behavior of 90% cocoa solid-containing chocolate; frictional behavior of molten chocolate samples and their lubricant film thickness; rationale behind the selection of the normal force in the licking stage; indenter motion in the *Z* direction for solid lubricity measurements; Fourier transform infrared analysis of 90% chocolate, the glass probe, and fat transfer film; motion of the top geometry in the *Z* direction for single-papilla and tongue-scale measurements; *in situ* tribomicroscopy of molten 90% chocolate and the corresponding video; and *in situ* tribomicroscopy of 90%-S saliva-mixed chocolate and the corresponding video (PDF)

## Notes

The data presented in this article will be openly available from the University of Leeds Data Repository: https://doi.org/10.5518/1279.
