# Supplementary material for: Insights into the Multiscale Lubrication Mechanism of Edible Phase Change Materials
Source: ACS Appl Mater Interfaces. 2023 Jan 12;15(3):3699–712. doi: 10.1021/acsami.2c13017 (PMC9880949; doi:10.1021/acsami.2c13017)
Supplement: Supplementary file 1 — am2c13017_si_001.pdf [file am2c13017_si_001.pdf]

Supporting information

Insights into the multiscale lubrication mechanism of edible phase change materials

Siavash Soltanahmadi[1], Michael Bryant[2], Anwesha Sarkar[1]*

[1] Food Colloids and Bioprocessing Group, School of Food Science and Nutrition, University of Leeds, UK

[2] Institute of Functional Interfaces, School of Mechanical Engineering, University of Leeds, UK

*Corresponding author:

Prof. Anwesha Sarkar

Food Colloids and Processing Group,

School of Food Science and Nutrition, University of Leeds, Leeds LS2 9JT, UK.

E-mail address: A.Sarkar@leeds.ac.uk (A. Sarkar).

CONTENT

S1. Details of composition and particle size of chocolate samples.

S2. Model saliva recipe.

S3. Rationale behind using the ratio of 1:1 for chocolate-S samples

S4. Image of the tongue-mimic tribological set-up.

S5. Rheological performance of chocolates measured using various geometries.

S6. Confocal micrographs of pristine and tribo-sheared chocolate samples.

S7. Frictional behaviour of 90% cocoa solid-containing chocolate.

S8. Frictional behaviour of molten chocolate samples and their lubricant film thickness

S9. Rationale behind selection of the normal force in the licking stage.

S10. Indenter motion in Z direction for solid lubricity measurements.

S11. Fourier-transform infrared analysis of 90% chocolate, the glass probe and fat transfer film.

S12. Motion of the top geometry in Z direction for single papilla and tongue scale measurements.

S13. *In situ* tribo-microscopy of molten 90% chocolate.

S14. Video showing the *in situ* tribo-microscopy of 90%.

S15. *In situ* tribo-microscopy of 90%-S saliva-mixed chocolate.

S16. Video showing the i*n situ* tribo-microscopy of 90%-S saliva-mixed chocolate.

S1. Details of composition and particle size of chocolate samples

Table S1| Details of composition and particle size of chocolate samples. Batch number and protein content in the chocolate samples as per the manufacturer, and measured specific surface area and the particle size (D[4,3]) (represented as means ± standard deviations) using static light scattering.

**Chocolates**	**70%**	**85%**	**90%**	**99%**
Batch number	L4570 214	L1610 311	L1630 38	L1570 302
Protein content (wt%)	9.5	12.5	10	15
Specific surface area (m^2 g^{-1})	1.225	1.151	1.163	1.091
Mean particle size, D [4,3] (μm)	4.26 ± 0.151	4.67 ± 0.168	4.44 ± 0.12	4.73 ± 0.010

S2. Model saliva recipe

Table S2| Model saliva recipe. The list of chemicals (chemical formula) and their concentrations used to produce the model saliva (S).[1]

Reagent	Concentration (g/L)
Sodium chloride (NaCl)	1.594
Ammonium nitrate (NH_4NO_3)	0.328
Potassium phosphate (KH_2PO_4)	0.636
Potassium chloride (KCl)	0.202
Potassium citrate ($K_3C_6H_5O_7.H_2O$)	0.308
Uric acid sodium salt ($C_5H_3N_4O_3Na$)	0.021
Urea (H_2NCONH_2)	0.198
Lactic acid sodium salt ($C_3H_5O_3Na$)	0.146
Porcine gastric Mucin Type II	3.0

S3. Rationale behind using the ratio of 1:1 for chocolate-S samples

The ratio of a food product to saliva influences the rheological and tribological properties of the resulting mixture [7]. Ratios between 1:1 and 200:0.5 have been used in studies of food and saliva mixtures to reflect the broad range of ratios in real oral conditions depending on the individual's physiology and the properties of food [7]. In this study the extreme ratio of 1:1 was used to replicate the last stage of oral processing, that is *bolus before swallowing*.

S4. Image of the tongue-mimic tribological set-up

A

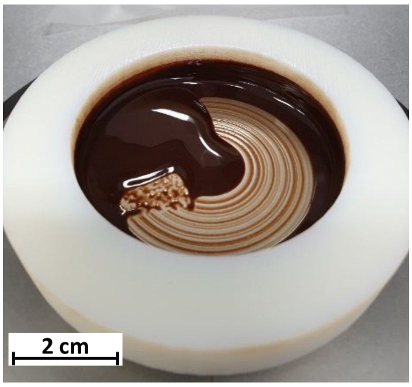

B

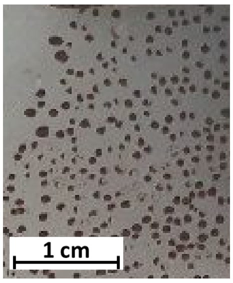

Figure S1| Image of the tongue-mimic tribological set-up. A) A macroscopic image taken after tribo-test at the tongue-mimic scale using the rheo-tribo setup showing the sweeping tracks of chocolates (brown circular tracks) created by the contact of hemisphere-shaped fungiforms on the biomimetic tongue elastomers. B) A macroscopic image of the surface of the biomimetic tongue elastomers showing the residuals of chocolates on the hemisphere-shaped fungiforms after the tribotests. (A) and (B) indicate an absence of direct contact between filiform-mimicked cylinders on the tongue-mimic elastomer and the bottom plate of the rheo-tribological set-up. Therefore, the origin of friction is the sum of shear stresses experienced by fungiform papillae.

S5. Rheological performance of chocolates measured using various geometries

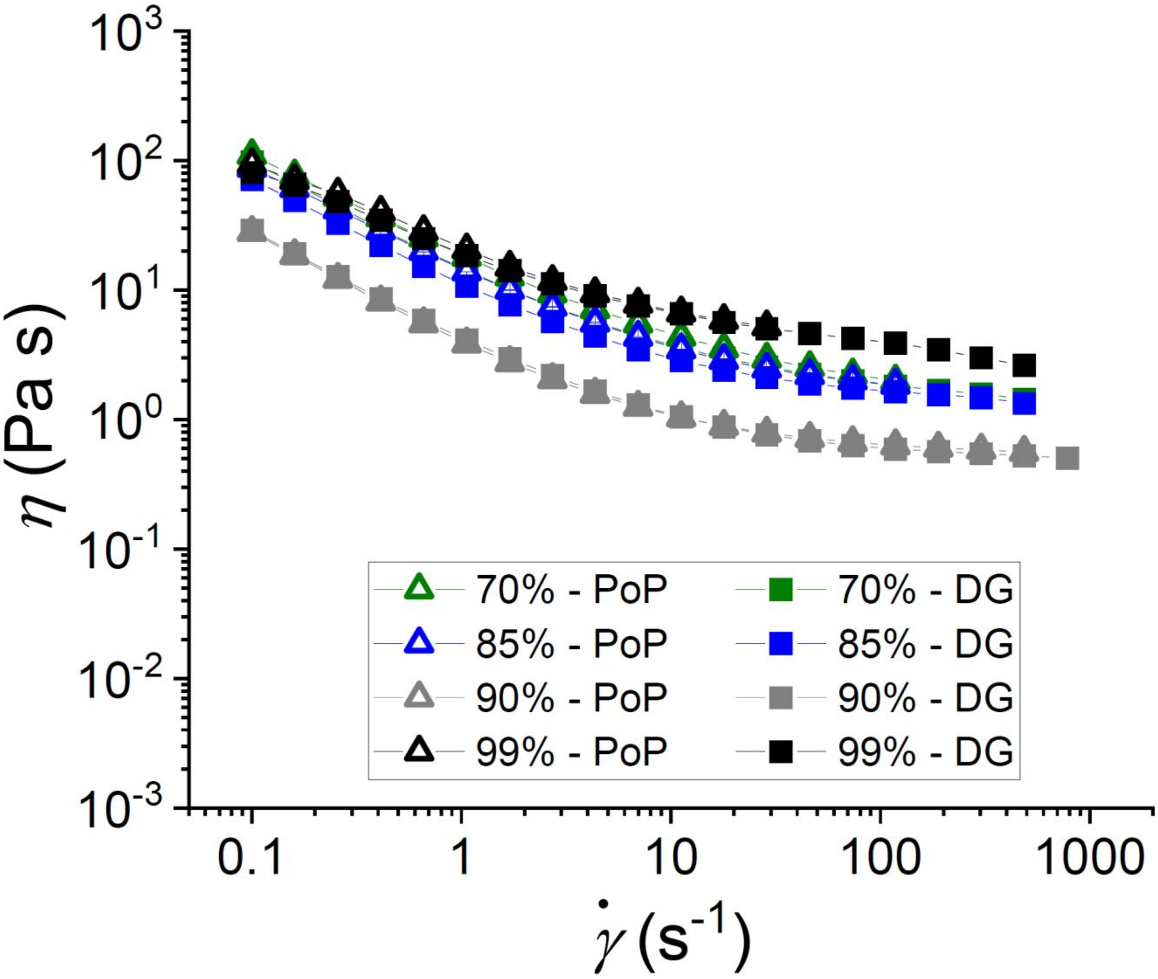

Figure S2| Rheological performance of chocolates measured using various geometries. Comparison of flow behaviour for chocolate samples using plate-on-plate (PoP) and double gap (DG) geometries. The DG geometry provides valid measurements until higher shear rates as compared to that of PoP, in PoP samples experienced an earlier slippage at higher shear rates. The viscosity values obtained with both geometries for each chocolate are almost identical at orally relevant shear rates (50 s^{-1}).

S6. Confocal micrographs of pristine and tribo-sheared chocolate samples

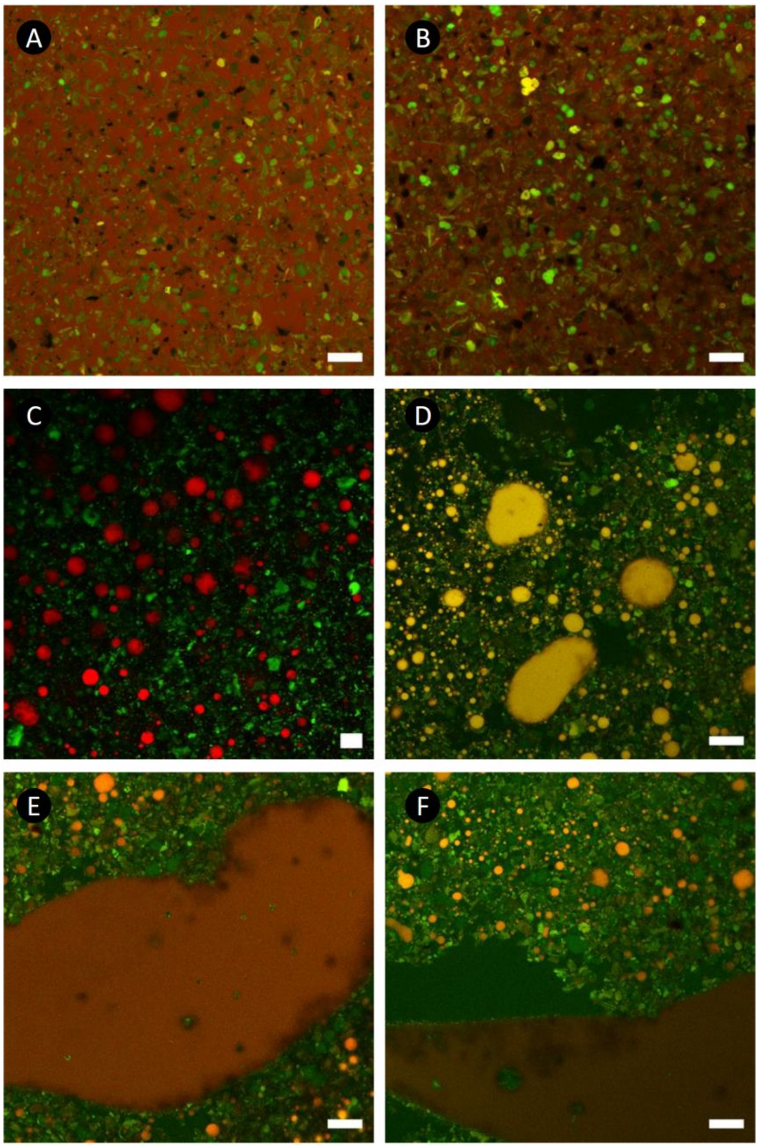

Figure S3| Confocal micrographs of pristine and tribo-sheared chocolate samples. Confocal laser scanning microscopic images of A) pristine un-sheared 70% chocolate, B) tribo-sheared 70% chocolate, C) pristine un-sheared 70%-S mixture, D) tribo-sheared 70%-S mixture, and E & F) tribo-sheared 90%-S mixture showing cocoa particle entrapment/ embedded into the coalesced fat droplets. The scale bars in the images represent 20 μm.

S7. Frictional behaviour of 90% cocoa-containing chocolate

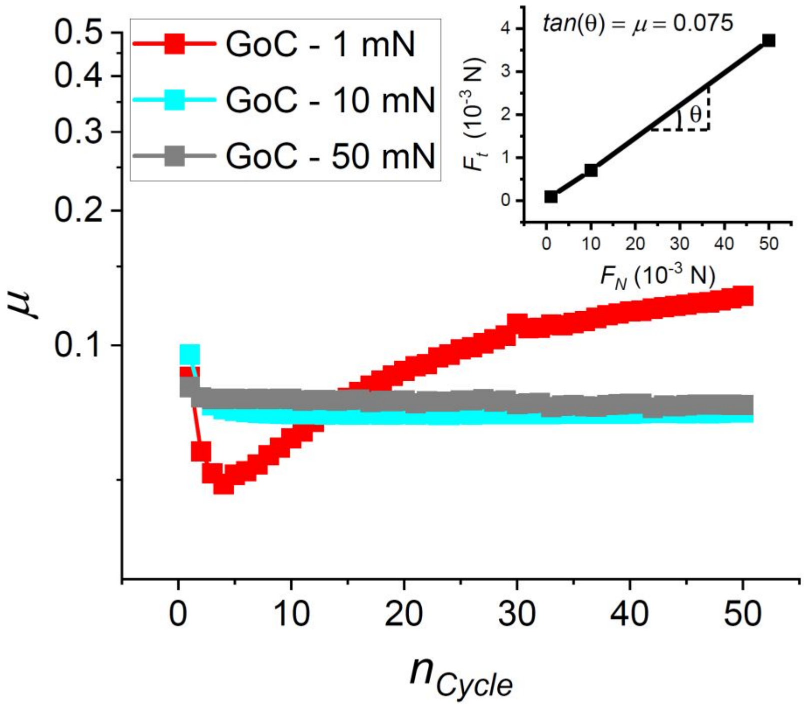

Figure S4| Frictional behaviour of 90% cocoa-containing chocolate. The coefficient of friction (μ) for the 90% chocolate in GoC configuration measured at three normal forces (F_N) of 1, 10 and 50 mN over fifty cycles of reciprocating sliding tribo-contact. The inset shows a linear correlation between F_N and the friction force (F_t) over a three-point fitting. The tangent to the F_N-F_t curve delivers the μ, which is almost identical to the μ obtained for the μ-n_{Cycle} plot at all F_N values (averaged over cycles 5 to 44).

A linear correlation between F_t and F_N was observed (**Figure S4**) which appears to be in agreement with the Bowden and Tabor theory [2, 3] (more information in the section **S6** of the SI). This suggests plastic deformation of surface irregularities and the top surface layer on the chocolate surfaces and that the Amontons's law, implying that the intimate contact area is independent of the apparent contact area [2], holds true for the solid-state licking of the chocolates. This can be expected when tribo-shear stresses are beyond the yield stress of the solid chocolates and implies that the friction behaviour in GoC/ GoC-S configuration is independent of the applied load for the parameters defined in this study.

S8. Frictional behaviour of molten chocolate samples and their lubricant film thickness

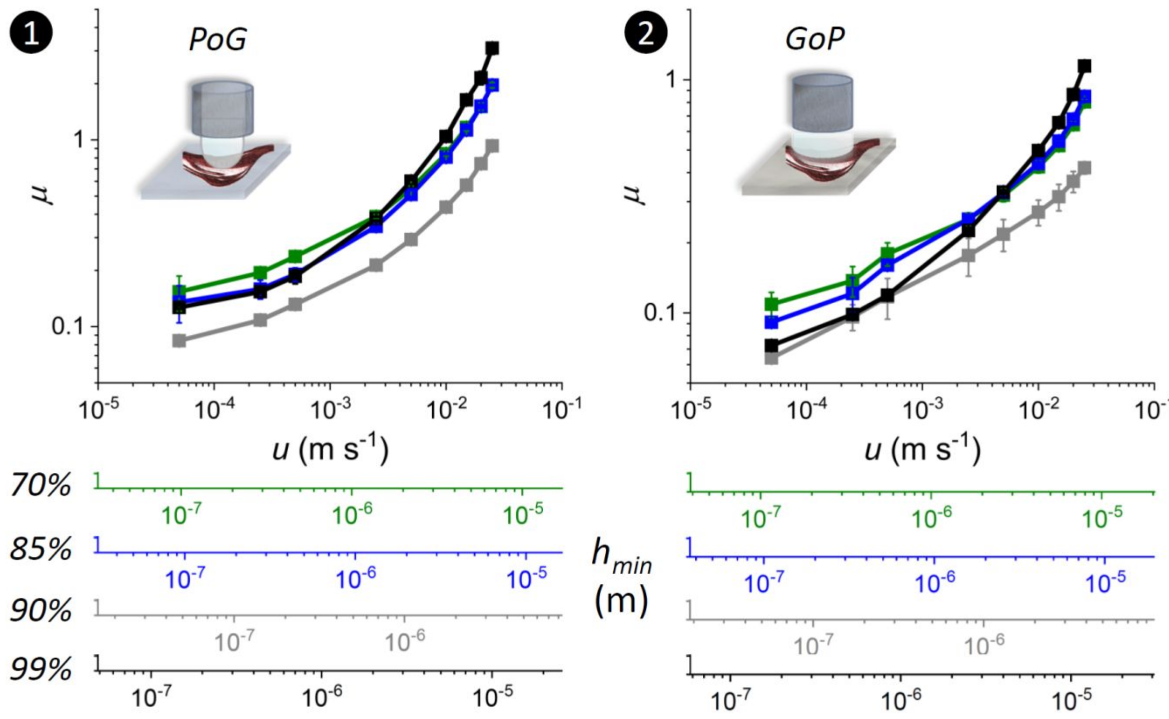

Figure S5| Frictional behaviour of molten chocolate samples and their lubricant film thickness. Coefficient of friction (μ) of the molten chocolates as a function of the entrainment speed (u) and theoretical minimum film thickness (h_{min}) in 1) PoG and 2) GoP configurations under the same contact pressure.

S9. Rationale behind selection of the normal force in the licking stage and the film thickness equation

During oral processing, a food product is confined between the tongue and the palate, while the softer tongue applies a load compressing the food against the harder palate. In our experimental setups, a contact load is applied by movement of a softer probe (representing a single papilla) or the softer tongue-mimic elastomer against harder surfaces, similar to the real oral conditions.

The rationale behind the selection of F_N and u for all tribological measurements is elucidated in the **Methods** section in the main document. In general, with higher solid particle content in chocolates, higher E values are expected [4, 5] and the storage conditions and testing parameters may also influence E [5, 6]. Using **Equations 2** and **3** (see **Methods** section in the main document) and considering reported values of E ~ 330 - 420 MPa for Lindt 70% - 90% [5] (obtained through a three-point bending test at 20°C) and a v = 0.4 (tensile tests) [6], P_{max} and $r_{contact}$ values of 3.4 – 3.9 MPa and 8 × 10^{-5} m were estimated, respectively, for the licking stage tribotests at F_N = 50 × 10^{-3} N.

Direct observations of wear tracks (the scar generated on the chocolate slabs during the tribotests, which is expected to be ~ 2 × $r_{contact}$) on chocolate surfaces showed wear track widths (*i.e.* 2 × r_{actual}, where r_{actual} is the real projected contact radius) in the order of 6 × 10^{-4} m. Therefore, the r_{actual} was substantially greater than $r_{contact}$. This suggested considerable plastic deformation of chocolates, which eventually results in significant drop in the actual contact pressure. This behaviour is expected since the Hertzian contact theory assumes elastic deformation of contacting surfaces under compression. Therefore, the actual contact pressure can be calculated from r_{actual} and F_N ($P = \frac{F_N}{\pi r_{actual}^2}$) which was determined to be ~ 180 kPa- that is comparable to P_{max} of tribotests at the single papilla-scale (**Figure 1.2**).

The relevant equation for the iso-viscous-elastic regime, which takes into account relatively low elastic moduli of the contacting materials (*i.e.* PDMS and tongue-mimic elastomer) and contact pressures in the orally-relevant tribo-conditions, was used to calculate the minimum film thickness (h_{min}) at the contact interface. The contact pressure is relatively low (in the order of kPa) in the soft contact of PDMS and the tongue-mimic surfaces and hence the iso-viscous regime was considered to account for the negligible influence of the contact pressure on the change of viscosity of the fluids (the chocolates and the mixtures of chocolates and the model saliva).

S10. Indenter motion in Z direction for solid lubricity measurements

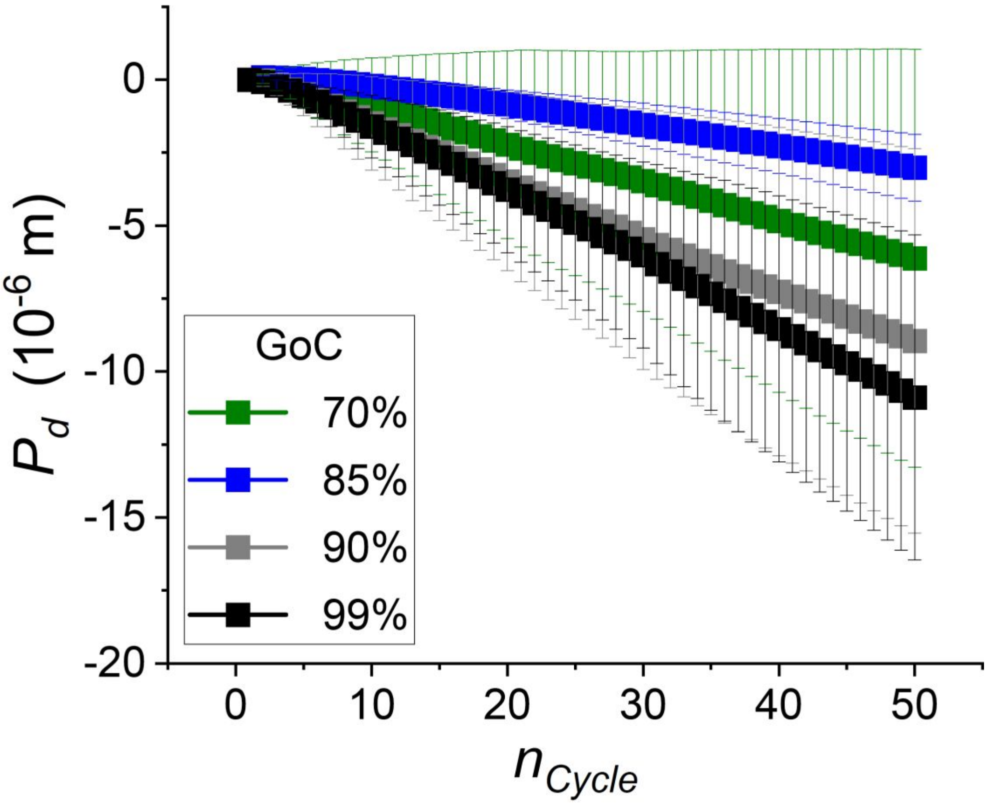

Figure S6| Indenter motion in Z direction for solid lubricity measurements. The graph shows movement of the indenter in Z direction (P_d) as a function of cycle number (n_{Cycle}) at an entrainment speed of 1 mm s^{-1} and normal force of 50 mN for GoC configuration. The negative values of P_d indicate penetration of the glass probe into the chocolate slabs and plastic deformation of the chocolate surfaces.

S11. Fourier-transform infrared analysis of 90% chocolate, the glass probe and fat transfer film.

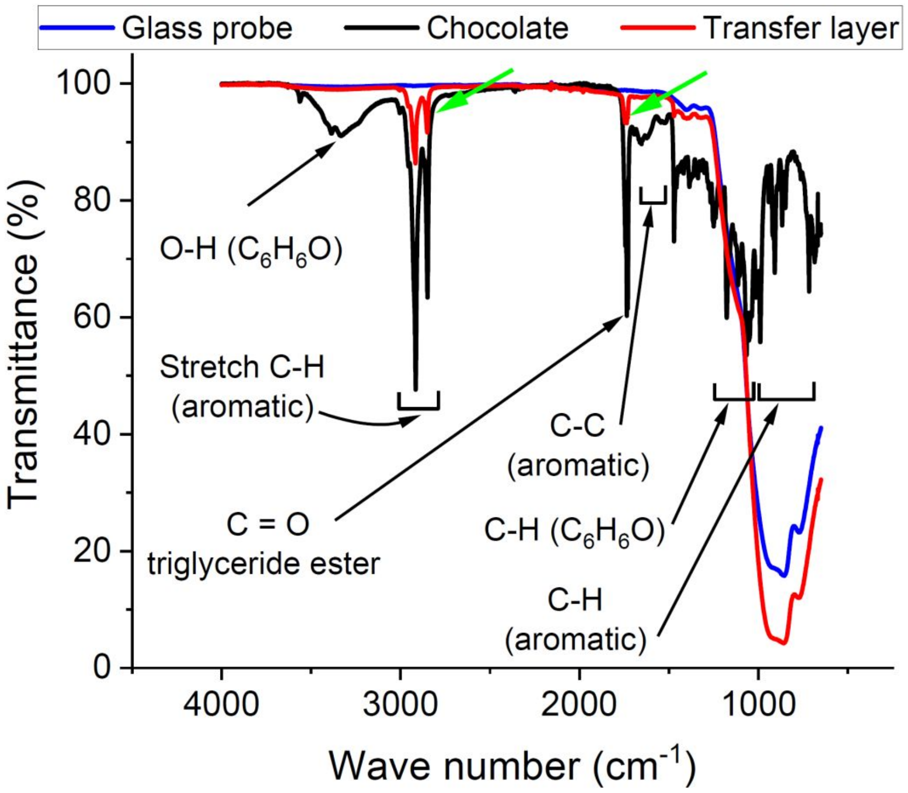

Figure S7| Fourier-transform infrared (FTIR) analysis of 90% chocolate, the glass probe and fat transfer film. Attenuated total reflection-FTIR spectra were obtained over wavelengths of 650–4000 cm^{-1}. The spectra for the borosilicate plano-convex glass used for the GoC/ GoC-S measurements and 90% chocolate, as reference materials, are shown in blue and black, respectively. The red spectrum was collected from the surface of the glass probe after tribo-contact in GoC configuration, referred to as *transfer film*. The assigned chemical bonds for the chocolate are shown in the figure using black arrows. The identified peaks in the spectrum of the transfer film were cross compared with the spectra from the reference spectra obtained from the Sigma library of FTIR and those of the chocolate and glass. As shown using green arrows, the aromatic C─H and C═O bands are clearly visible in the spectrum of the transfer film indicating the presence of a fat film on the glass surface (transferred from the chocolate) after the tribo-contact.

S12. Motion of the top geometry in Z direction for single papilla and tongue scale measurements

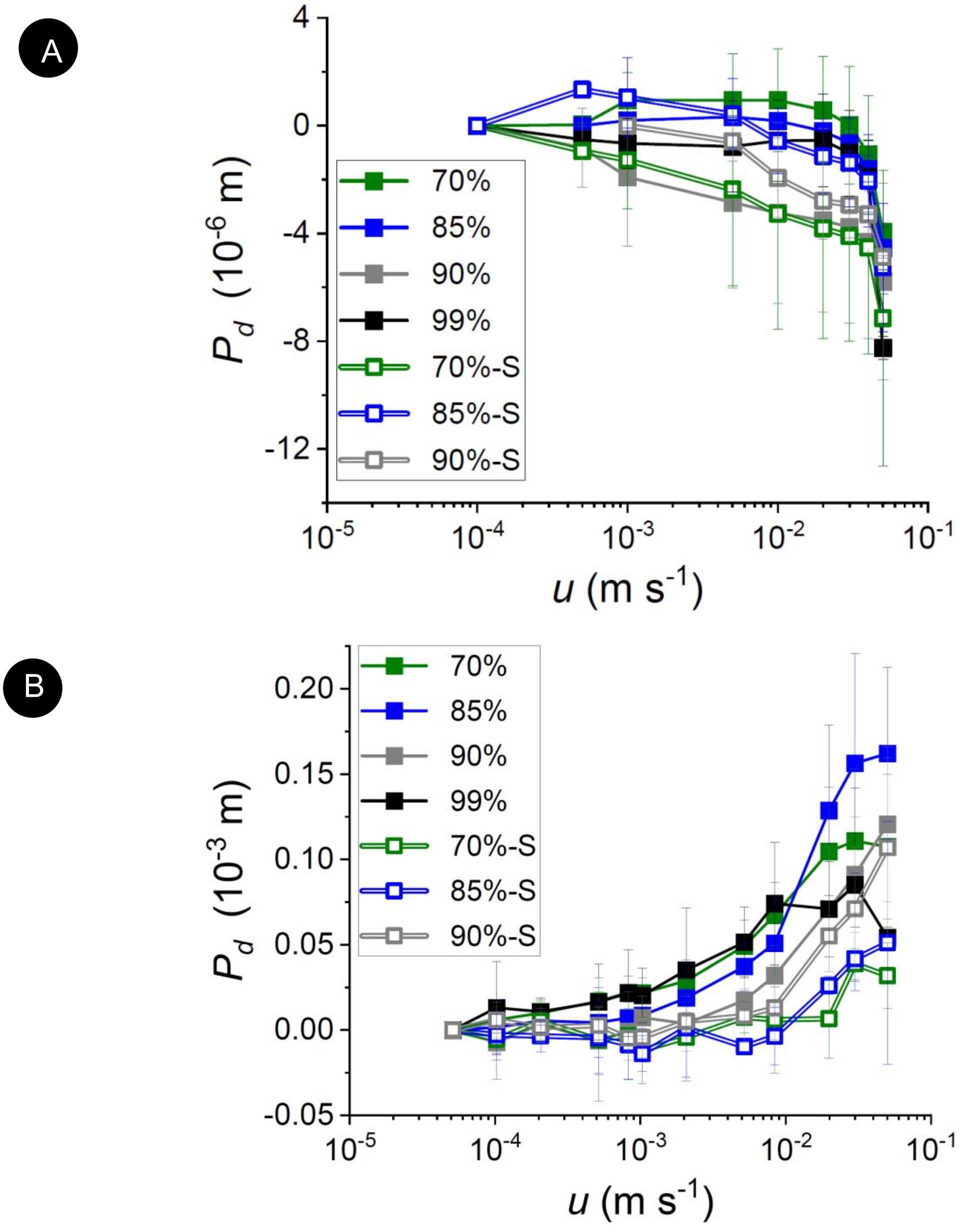

Figure S8| Motion of the top geometry in Z direction for single papilla and tongue scale measurements. Movement of the top geometries in Z direction (P_d) as a function of entrainment speed (u) at A) single papilla-scale (PoG configuration) and B) tongue-scale. The P_d values dropped to negative values as u increased, in A) corroborating our hypothesis of impaired bridging effect. Increased P_d values in B) with increased u values indicate separation of contact surfaces by chocolate or chocolate-S films.

S13. *In situ* tribo-microscopy of molten 90% chocolate.

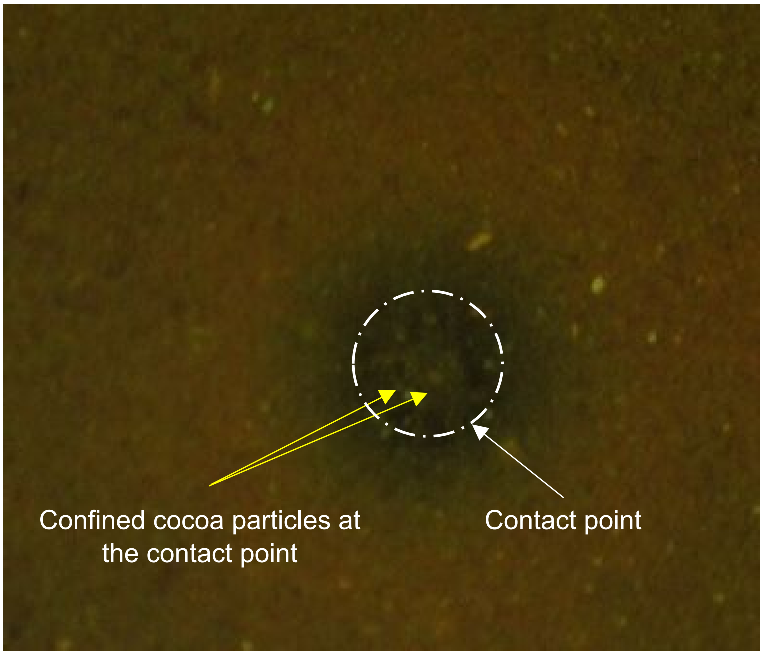

Figure S9| A snapshot showing the in situ tribo-microscopy of 90%. The auto-florescent illumination of flavonoids in cocoa solids brought about green contrast in the figure at the circular contact point. The irregular-shaped particles, shown using yellow arrows, confirm the confinement of particles at the contact interface.

S14. Video showing the *in situ* tribo-microscopy of molten 90% chocolate.

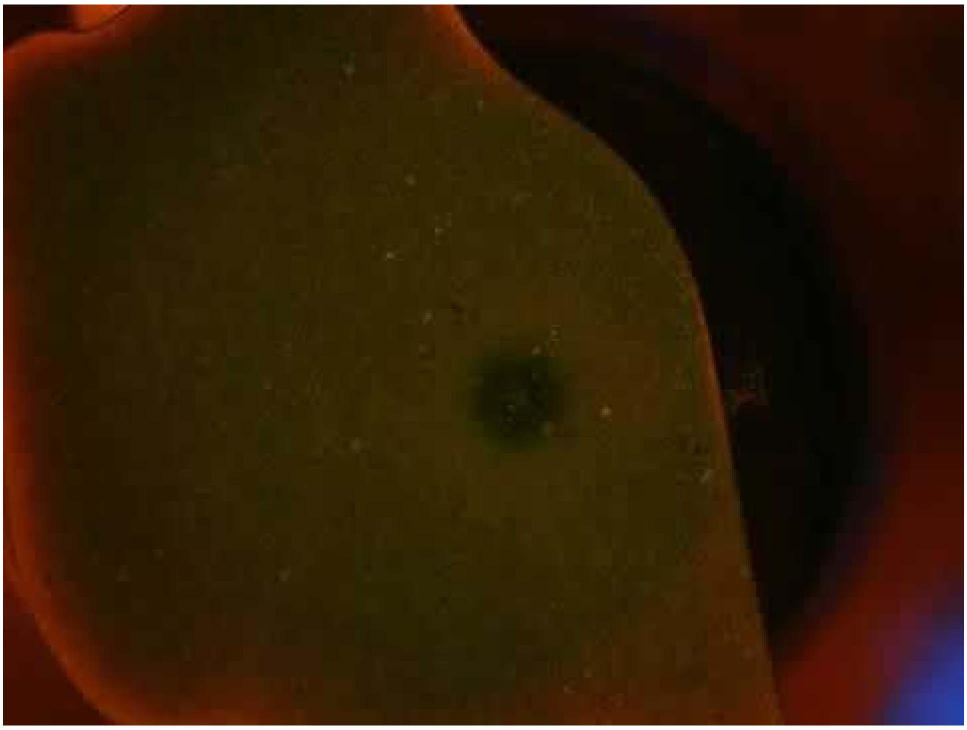

Video SV1| Video showing the *in situ* tribo-microscopy of 90%.

S15. *In situ* tribo-microscopy of 90%-S saliva-mixed chocolate.

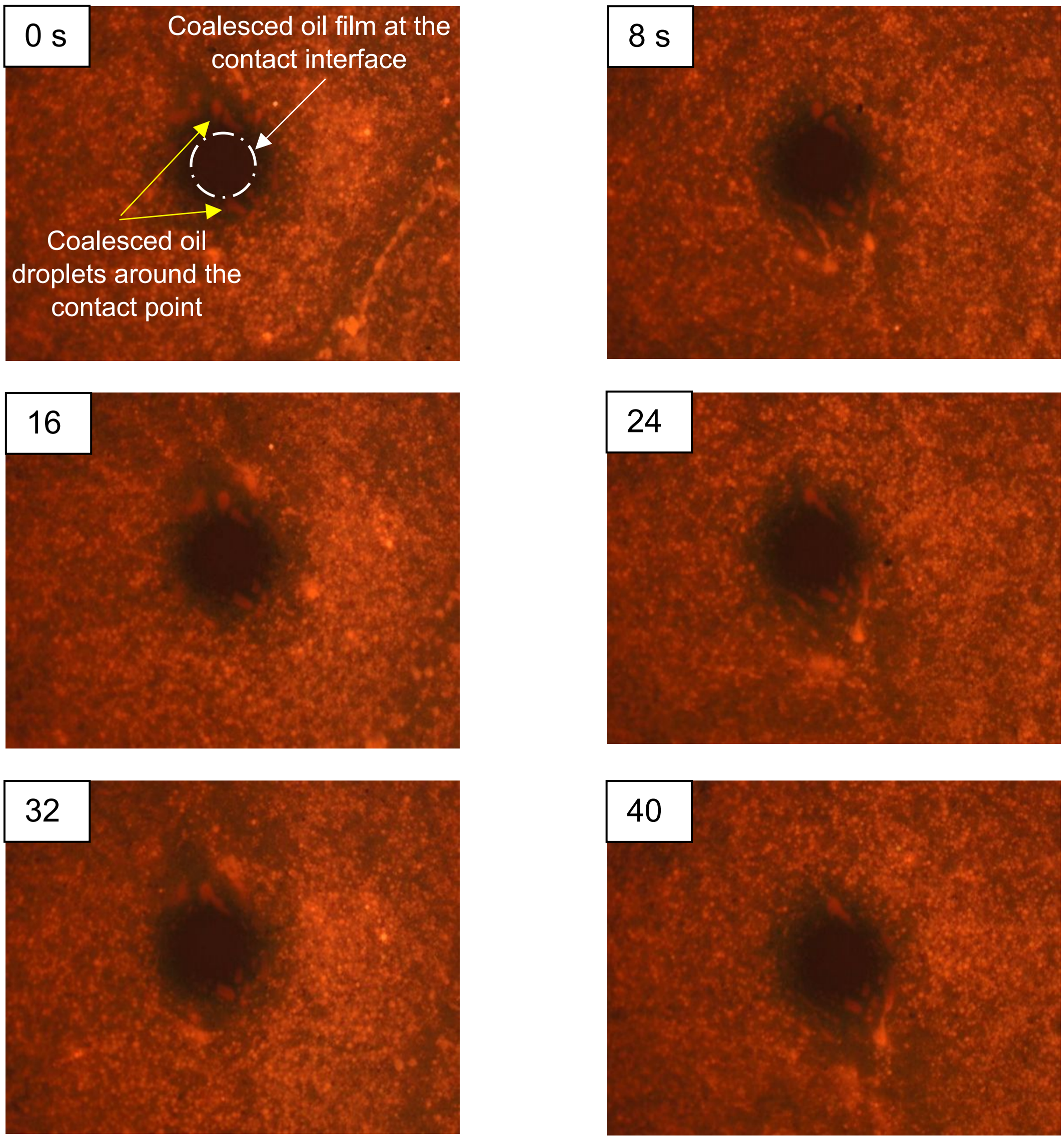

Figure S10| Snapshots showing the in situ tribo-microscopy of stained 90%-S after timer intervals of 8 s. The red droplets in the figure show the cocoa butter oil droplets around the darker brown circular contact point. The irregular-shaped coalesced oil droplets around the contact point are shown using yellow arrows in the first snapshot. Disappearance (splitting) and evolution (coalescence) of oil droplets can be tracked between relative times of: 0 - 40 s

S16. Video showing the *in situ* tribo-microscopy of 90%-S saliva-mixed chocolate.

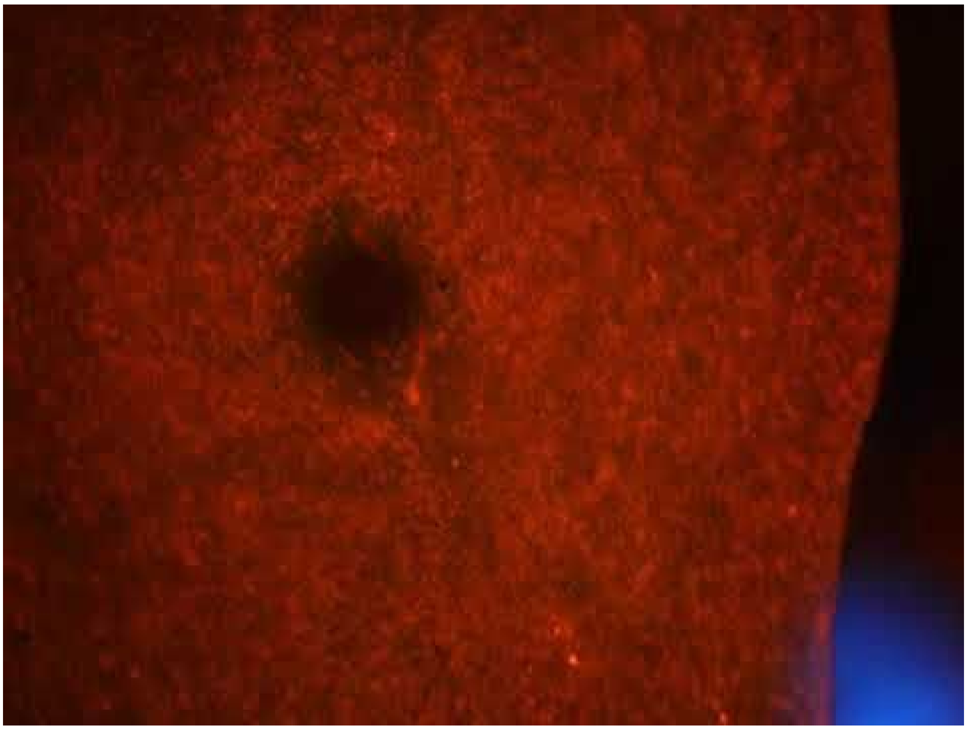

Video SV2| Video showing the *in situ* tribo-microscopy of 90%-S saliva-mixed chocolate.

References:

(1) Sarkar, A.; Goh, K. K. T.; Singh, H. Colloidal stability and interactions of milk-protein-stabilized emulsions in an artificial saliva. *Food Hydrocolloids* **2009**, *23* (5), 1270-1278. DOI: https://doi.org/10.1016/j.foodhyd.2008.09.008.
(2) Bowden, F. P.; Tabor, D. J. P. o. t. R. S. o. L. S. A. M.; Sciences, P. The area of contact between stationary and moving surfaces. **1939**, *169* (938), 391-413.
(3) Liang, X. M.; Xing, Y. Z.; Li, L. T.; Yuan, W. K.; Wang, G. F. An experimental study on the relation between friction force and real contact area. *Scientific Reports* **2021**, *11* (1), 20366. DOI: 10.1038/s41598-021-99909-2.
(4) Afoakwa, E. O.; Paterson, A.; Fowler, M. Effects of particle size distribution and composition on rheological properties of dark chocolate. *European Food Research and Technology* **2008**, *226* (6), 1259-1268. DOI: 10.1007/s00217-007-0652-6.
(5) Zhao, H.; Li, B.; James, B. J. Structure-fracture relationships in chocolate systems. *LWT* **2018**, *96*, 281-287. DOI: https://doi.org/10.1016/j.lwt.2018.05.045.
(6) Tremeac, B.; Hayert, M.; Le-Bail, A. Mechanical properties of Tylose gel and chocolate in the freezing range. *International Journal of Refrigeration* **2008**, *31* (5), 867-873. DOI: https://doi.org/10.1016/j.ijrefrig.2007.10.005.
(7) Laguna, L.; Fiszman, S.; Tarrega, A. Saliva matters: Reviewing the role of saliva in the rheology and tribology of liquid and semisolid foods. Relation to in-mouth perception. *Food Hydrocolloids* **2021**, *116*, 106660. DOI: https://doi.org/10.1016/j.foodhyd.2021.106660.
